# Psychometric Performance of Generic Childhood Multi-Attribute Utility Instruments in Preterm and Low Birthweight Populations: A Systematic Review

**DOI:** 10.3390/children10111798

**Published:** 2023-11-10

**Authors:** Joseph Kwon, Corneliu Bolbocean, Olu Onyimadu, Nia Roberts, Stavros Petrou

**Affiliations:** 1 Nuffield Department of Primary Care Health Sciences, University of Oxford, Oxford OX2 6GG, UK; joseph.kwon@phc.ox.ac.uk (J.K.); corneliu.bolbocean@phc.ox.ac.uk (C.B.); olu.onyimadu@phc.ox.ac.uk (O.O.); 2 Bodleian Health Care Libraries, University of Oxford, Oxford OX3 9DU, UK; nia.roberts@bodleian.ox.ac.uk

**Keywords:** preterm population, health utility, psychometric performance

## Abstract

Background: Individuals born preterm (gestational age < 37 weeks) and/or at low birthweight (<2500 g) are at increased risk of health impairments from birth to adulthood. This review aimed to evaluate the psychometric performance of generic childhood-specific or childhood-compatible multi-attribute utility instruments (MAUIs) in preterm and/or low birthweight (PLB) populations. Methods: Searches covered seven databases, including studies that targeted childhood (aged < 18 years) and/or adult (≥18 years) PLB populations; provided psychometric evidence for generic childhood-specific or compatible MAUI(s) (any language version); and published in English. Eighteen psychometric properties were evaluated using a four-part criteria rating system. Data syntheses identified psychometric evidence gaps and summarised the psychometric assessment methods/results. Results: A total of 42 studies were included, generating 178 criteria rating outputs across four MAUIs: 17D, CHSCS-PS, HUI2, and HUI3. Moreover, 64.0% of outputs concerned the HUI3 MAUI, and 38.2% related to known-group validity. There was no evidence for five psychometric properties. Only 6.7% of outputs concerned reliability and proxy–child agreement. No MAUI outperformed others across all properties. The frequently applied HUI2 and HUI3 lacked content validity evidence. Conclusions: This psychometric evidence catalogue should inform the selection of MAUI(s) suited to the specific aims of applications targeting PLB populations. Further psychometric research is warranted to address the gaps in psychometric evidence.

## 1. Background

Individuals born preterm (gestational age < 37 weeks) and/or at low birthweight (<2500 g) are at an increased risk of a range of physical, motor, cognitive, psychosocial, and behavioural problems that persist from the neonatal period into adulthood [1,2,3,4,5,6,7]. These problems likely impair the individuals’ health-related quality of life (HRQoL), which consists of multiple dimensions of health perceived by individuals to impact their well-being or quality of life [8,9,10]. They are likewise associated with lower health utilities, anchored on a scale with 0 = dead and 1 = full health. Health utilities are typically measured using a multi-attribute utility instrument (MAUI) containing a pre-specified multidimensional health classification system and one or more value set(s) that reflect(s) the stated preferences (typically of a representative sample of the general adult population) for the health states generated by the classification system [11,12,13]. The health impairments of preterm and/or low birthweight (PLB) individuals can thus be measured and valued using an MAUI, which can be either generic in its application (i.e., applicable to all disease areas and populations; e.g., EQ-5D-3L [14]) or condition-specific (e.g., CP-6D for cerebral palsy [15]).

The health utilities generated using MAUIs can serve as inputs into economic evaluations that compare the costs and consequences of alternative interventions targeting PLB populations [16]. Specifically, cost-utility analysis is a form of economic evaluation that uses the quality-adjusted life year, which combines utility values for health states with the length of time spent in those states, as the primary health outcome [16]. Cost-utility analysis has been recommended by several national healthcare decision-makers to inform resource allocation across disease areas and populations based on cost-effectiveness [17,18,19,20]. Cost-utility analyses of interventions that target PLB populations thus require the measurement and valuation of both the economic costs [21] and the health utilities [22] associated with the health sequelae of PLB from birth into childhood (aged < 18 years) and potentially adulthood (aged ≥ 18 years).

There are nevertheless key challenges for measuring health utilities in childhood populations in general. First, rapid biopsychosocial development during childhood means that the relevant dimensions of HRQoL are likely to vary by childhood age [23]. Second, the wording and format (e.g., use of pictures) of the measurement instrument should be tailored to the comprehension level and attention span of the target childhood age group [24,25]. Third, there is a frequent need to rely on proxy respondents such as parents when outcomes are assessed in children [24], which generates uncertainty regarding the level of agreement between child self-report and proxy-report [26,27]. These issues are particularly relevant for PLB populations that have a higher prevalence of cognitive and attention impairments during childhood and beyond [28,29]. Given these challenges, a range of MAUIs has been developed with childhood-specific (i.e., applicable only in childhood populations) or childhood-compatible (i.e., applicable in childhood and adult populations) classification systems and formats, as well as preference-based value sets derived from childhood and/or adult samples [13]. Specifically, a recent systematic review identified 14 generic MAUIs that are specific to or compatible with childhood populations (see full list under ‘Methods’) [25].

Psychometrics concerns the performance of measurement scales and is applied in healthcare to develop scientifically rigorous patient-reported outcome measures (PROMs) of health, including MAUIs [30,31,32]. Key psychometric properties include content validity, reliability, construct validity, responsiveness, and patient and investigator burden (acceptability) [24,26,31,32,33,34]. Each of these properties requires unique tests and criteria and contributes to minimum scientific standards for the use of a given PROM or MAUI in research and decision-making [33]. To be considered for use, an MAUI should demonstrate acceptable performance across all properties included in the minimum standard set [35]. Importantly, the content of such standards varies across research and decision-making settings and target populations. The selection of PROMs for a randomised controlled trial (RCT), for example, is likely to prioritise the psychometric property of responsiveness, i.e., the ability of the PROM to identify change in the underlying health construct affected by the trialled intervention (relative to its comparator) [33].

A recent systematic review identified and synthesised evidence for the psychometric performance of 14 generic MAUIs specific to or compatible with childhood [35]. The aim of this previous review was to create a comprehensive catalogue of published psychometric evidence, covering all general and clinical childhood populations, to identify evidence gaps for further psychometric research. The review included 372 studies and generated a catalogue of 2153 criteria rating outputs (which are outcomes from the review’s evaluation of the psychometric assessments of the included studies). No MAUI consistently outperformed others across all 18 psychometric properties considered by the review (see ‘Methods’ for a list of these properties).

Notably, the aggregated reporting of the identified psychometric evidence by the above review across all childhood population groups precluded any judgment on the relative performance of the MAUIs for specific clinical populations such as PLB populations. This specific focus is important given that the minimum standard set for psychometric performance likely varies across populations. To that end, the current study aims to conduct a systematic review of the published psychometric evidence of the 14 childhood-specific or compatible MAUIs in PLB populations. These MAUIs are potentially applicable to both children and adults; hence, studies that applied them in adult PLB populations were also included. This allows an evaluation of whether the psychometric performance of the MAUIs varies across childhood and adulthood. Previous systematic reviews of HRQoL in the PLB population focused on HRQoL differences between PLB groups and term-born and/or normal BW controls and not on the psychometric aspects of the MAUIs used [22,36]. The objectives of this systematic review are to:

(1) Create a catalogue of evaluated psychometric evidence that can aid in the selection of generic childhood-specific or childhood-compatible MAUIs for application in PLB populations;
(2) Identify gaps in psychometric evidence to inform future psychometric research in this population;
(3) Summarise the commonly used psychometric assessment methods and the relative psychometric performance of instruments by property.

## 2. Methods

A pre-specified protocol outlining the systematic review methods was developed and registered with the Prospective Register of Systematic Reviews (CRD42023428176). The PRISMA 2020 guideline was followed [37]: see the Supplementary Information for the PRISMA checklist. Figure S1 in the Supplementary Information graphically illustrates the systematic review method and objectives.

### 2.1. Data Sources and Study Selection

The database searches aimed to identify studies targeting PLB populations that provide evidence for the psychometric performance of one or more of the following 14 generic childhood-specific or childhood-compatible MAUIs identified and evaluated in a systematic review [25]:

- 16D: 16-dimensional health-related measure [38]
- 17D: 17-dimensional health-related measure [39]
- AHUM: Adolescent Health Utility Measure [40]
- AQoL-6D Adolescent: Assessment of Quality of Life, 6-Dimensional, Adolescent [41]
- CH-6D: Child Health—6 Dimensions [42]
- CHSCS-PS: Comprehensive Health Status Classification System—Preschool [43]
- CHU9D: Child Health Utility—9 Dimensions [44,45]
- EQ-5D-Y-3L: EuroQoL 5 Dimensional questionnaire for Youth 3 Levels [46]
- EQ-5D-Y-5L: EQ-5D-Y 5 Levels [47]
- HUI2: Health Utilities Index 2 [48]
- HUI3: Health Utilities Index 3 [49]
- IQI: Infant health-related Quality of life Instrument [50]
- QWB: Quality of Well-Being scale [51,52]
- TANDI: Toddler and Infant health related quality of life instrument [53,54].

Of the above, the HUI2, HUI3, and QWB are childhood-compatible, and the rest are childhood-specific [25].

An information specialist (NR) guided the database choice and designed the search strategy to maximise the coverage of studies that applied MAUIs in general and clinical populations. Seven databases were searched from the database’s inception to 26 April 2023: Medline, Embase, PsycInfo, EconLit, CINAHL, Scopus, and Science Citation Index. The search strategies are shown by database in Tables S1–S7 in the Supplementary Information. Three co-authors (JK, OO, and CB) independently reviewed the titles and abstracts and then the full texts using Covidence [55]. An article that received two approvals proceeded to the next stage (title/abstract, full text, and then data extraction). Disagreements were referred to SP for arbitration.

There were three main inclusion criteria: (1) The study contained evidence for at least one psychometric property (see Section 2.3 below for a list of properties) of one or more of the 14 MAUIs in any language version. (2) It targeted a PLB population (gestational age < 37 weeks and/or birthweight < 2500 g) and/or relevant proxy respondents. (3) It was published in English. Adult populations aged ≥ 18 years were included as long as the above criteria were met. Studies that did not directly assess psychometric performance but contained relevant psychometric evidence were included as ‘indirect’ assessment studies (e.g., RCT in a PLB population for evidence on responsiveness).

The exclusion criteria were: (i) the study used one of the MAUIs as a criterion standard to validate a new instrument; (ii) the study targeted patients with specific diseases common in preterm birth (e.g., cerebral palsy) rather than the PLB population more generally; (iii) the study developed and validated value sets for health utility derivation without assessing or providing evidence of the psychometric properties of the health utilities.

### 2.2. Data Extraction

Data from the included studies were extracted by JK, and 20% was independently extracted by OO and CB. The following data fields were extracted and stored in Excel: (i) bibliography; (ii) study country(ies); (iii) study design—e.g., RCT; (iv) direct or indirect assessment psychometric properties; (v) psychometric property(ies) assessed; (vi) methodological issues for psychometric assessment; (vii) MAUI(s) assessed—any language version(s); (xiii) MAUI component(s) assessed—e.g., utility score after valuation, dimension level response; (ix) value set derivation country and population; (x) respondent type(s)—self-report and/or proxy report; (xi) administration mode(s)—e.g., with interviewer; (xii) study population clinical characteristics including gestational age and birthweight; (xiii) target and sample age—e.g., mean, range—and proportion of females; (xiv) target and actual sample size; (xv) intervention(s) assessed (if relevant).

### 2.3. Evaluation and Data Synthesis

Table S8 in the Supplementary Information defines the psychometric properties evaluated: internal consistency, test–retest reliability, inter-rater reliability, inter-modal reliability, proxy–child agreement, content validity, structural validity, cross-cultural validity, known-group validity, hypothesis testing, convergent validity, discriminant validity, empirical validity, concurrent validity, predictive validity, responsiveness, acceptability, and interpretability. The properties are contained in established standards developed and used by stakeholders involved in the psychometric performance of PROMs, including the COnsensus-based Standards for the selection of health Measurement INstruments (COSMIN) checklist [56,57,58], the International Society for Quality of Life Research guideline [33], the Food and Drug Administration guideline [31], and the Medical Outcomes Trust guidelines [34].

This review evaluated the psychometric assessments conducted by the included primary studies in terms of their assessment methods and the resulting psychometric evidence. Table S8 describes a four-part criteria rating for evaluating each property (a three-part rating for interpretability), which produced a criteria rating output per assessment. An output of ‘+’ indicates psychometric evidence consistent with the primary study’s a priori hypothesis according to its clinical and/or psychometric expectation; ‘±’ is partially consistent, and ‘−’ indicates no evidence or contrary evidence to the a priori hypothesis. An output of ‘?’ indicates the poor quality of assessment design and methods (e.g., insufficient sample size for statistical power, inappropriate statistical technique) that precluded the sufficient evaluation of psychometric performance. Each property also had unique assessment method requirements [57].

Comprehensive evaluation required context-specific judgements which are reported in the online Excel Supplementary File on a case-by-case basis. Differences in the primary study’s a priori hypothesis were a key source of between-context variation. For example, one study expected HRQoL between PLB adults and full-term/normal birthweight controls to be similar [59], while another expected lower HRQoL for PLB adults [60]. Where a priori hypotheses were clearly stated, these were followed by the review. In a study that conducted a known-group validity assessment for the HRQoL difference between the PLB group and full-term/normal birthweight controls without stating an a priori hypothesis, it was assumed that lower HRQoL is expected for the PLB group.

The criteria rating outputs were synthesised to address the three review objectives:

(1) Create a catalogue of evaluated psychometric evidence. The online Excel file serves as the main catalogue wherein the criteria rating outputs are tabulated and the main rationale for each rating. More condensed catalogues are presented in this manuscript.
(2) Identify gaps in psychometric evidence. Two aspects were defined as evidence gaps: (i) no criteria rating output available for an MAUI for a property and (ii) no criteria rating output available or where available no ‘+’ output. The number of these cases was computed for the whole evidence base and for a subset of evidence involving PLB adult populations.
(3) Summarise the psychometric assessment methods and performance of instruments by property. The psychometric assessment methods used by the included studies were described by property. The relative performance of MAUIs was compared by property, using the proportion of ‘+’ as the performance metric and also considering the absolute number of outputs.

## 3. Results

### 3.1. Search Results

**Figure 1:**
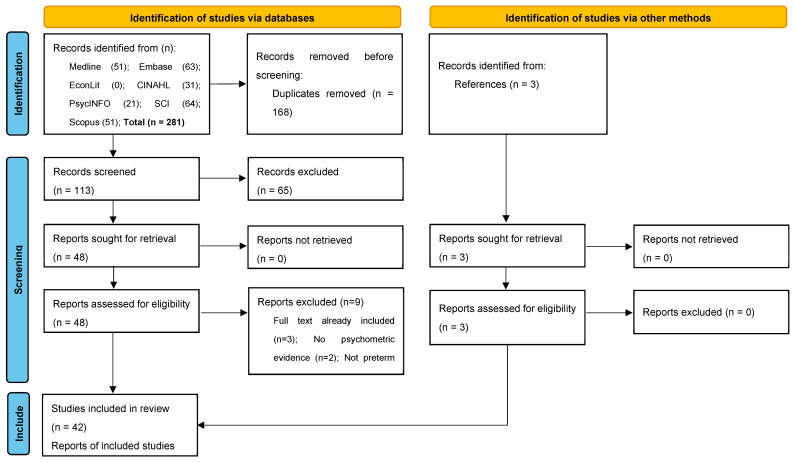
Flow diagram of the preferred reporting items for systematic reviews and meta-analyses.

Figure 1 presents the PRISMA flow diagram. After the screening of titles/abstracts and full texts, 42 studies were included in the systematic review. Nine studies excluded at the full-text screening stage are listed in Table S9 in the Supplementary Information alongside the main reason for their exclusion.

### 3.2. Characteristics of Included Studies

Table 1 shows the characteristics of the 42 included studies. Only nine countries were represented, all of which were high-income except for one (Jamaica) [61]. A total of 15 cohorts of PLB populations were represented. Seven cohorts were analysed by 34 studies with multiple studies per cohort. Figure S2 in the Supplementary Information illustrates the target age of the 34 studies, grouped by the seven cohorts. Only five studies conducted longitudinal analyses of repeated measurements [62,63,64,65,66]. Peart and colleagues [67] analysed the change in HRQoL at age eight across the multiple cohorts of the Victorian Infant Collaborative Study (infants recruited in 1991–1992, 1997, and 2005). Only four MAUIs, 17D, CHSCS-PS, HUI2, and HUI3, were applied by the 42 studies included in the systematic review. The HUI3 was used by 29 of 42 (69.0%) studies, six of which applied the HUI3 alongside the HUI2. Fifteen (35.7%) studies used the HUI2, three used the 17D, and one used the CHSCS-PS.

Eight studies were judged by the review authors as having a direct aim of assessing the psychometric performance of MAUI(s) [43,68,69,70,71,72,73,74]. Bolbocean and colleagues [68] compared the relative performances of the HUI3 and adult-specific SF-6D in capturing health utility differences between PLB and full-term/normal birthweight groups. Feeny and colleagues [69] assessed the agreement between the HUI2/3 utility scores generated by the application of an available value set and health utility directly measured using a standard gamble. Roberts and colleagues [70] estimated the sensitivity and specificity of the HUI2 utility score in screening for disability. Saigal and colleagues (1994) [71] explored whether adding two dimensions to the HUI2 (behaviour and general health) and removing the fertility dimension made the instrument more suitable for extremely low birthweight (ELBW) children. Three studies evaluated the agreement between the responses of PLB children and parents for the HUI2 [72] and HUI3 [73,74]. Saigal and colleagues (2005) [43] reported on the development of the CHSCS-PS involving preschool children who exhibited a very low birthweight (VLBW), a normal birthweight, or who were diagnosed with cerebral palsy.

### 3.3. Characteristics of Psychometric Assessments

The 42 included studies, in total, conducted 178 psychometric assessments. Table 2 summarises the characteristics of these assessments. See Table S10 in the Supplementary Information for the criteria rating outputs generated from these assessments by study. The eight direct assessment studies (19.0% of studies) conducted a proportionately larger number of psychometric assessments (25.8% of assessments). Relatively even numbers of assessments targeted extremely preterm and/or extremely low birthweight (EP/ELBW) (53.4%) and very preterm and/or very low birthweight (VP/VLBW) (44.9%) populations. Infants and preschool children aged < 5 years were underrepresented, with only seven assessments from one study [43]. Around one-fifth (19.7%) of assessments targeted PLB adults, while 15 (8.4%) assessments from three studies [62,63,64] included both children and adults. Around one-fifth (18.5%) of assessments involved self-reports by PLB individuals but with proxy support for the severely impaired subgroup. Such support was required in 14 of 35 (40.0%) assessments targeting adults.

### 3.4. Psychometric Evidence Gaps

For the second review objective, Table 3 summarises the availability of psychometric evidence from all included studies, disaggregated by target age group. It shows the number of criteria rating outputs by MAUI and psychometric property as well as the percentage of outputs evaluated as ‘+’. Around two-thirds (64.0%) of outputs from all studies concerned the HUI3 and around two-fifths (38.2%) concerned known-group validity. There was no evidence for structural validity, cross-cultural validity, discriminant validity, empirical validity, and predictive validity. There was no output of ‘+’ for internal consistency, test–retest reliability, inter-rater reliability, inter-modal reliability, convergent validity, responsiveness, and interpretability. Evidence for the HUI3 covered the greatest number of properties (eight) from all studies, but evidence for the CHSCS-PS covered the greatest number of properties for which there was at least one ‘+’ output (five vs. three for HUI3).

**Table 1:** Characteristics of included studies.

#	Reference	Country	Cohort	Population Characteristics	Target Age	Evidence ^1^	Sample Size	MAUI ^2^	Respondent
1	Achana 2022 [75]	UK	EPICure2	EP (<27 weeks); normal term controls from mainstream schools matched by age and sex where possible	11	Indirect	PLB 200; C 143	HUI2; HUI3	Parents or other
2	Baumann 2016 [64]	Germany	BLS	VP (<32 weeks) and/or VLBW (<1500 g); normal term/BW controls from same birth hospitals matched by sex and family SES	13, 26 (RM)	Indirect	PLB 190; C 201	HUI3	PLB; parents
3	Bolbocean 2023 [68]	Australia, Germany	BLS, VICS 1991-2	VP (<32 weeks) and/or VLBW (<1500 g); normal term/BW controls	BLS 26, VICS 18	Direct	PLB 558; C 491	HUI3	PLB
4	Bolbocean 2023b [76]	Australia, Germany, Ireland, UK	BLS, EPICure, VICS 1991-2	VP (<32 weeks) and/or VLBW (<1500 g); normal term/BW controls	BLS 26, EPICure 19, VICS 18	Indirect	PLB 527; C 423	HUI3	PLB
5	Breeman 2017 [77]	Germany, The Netherlands	BLS, POPS	VP (<32 weeks) and/or VLBW (<1500 g)	BLS 26, POPS 28	Indirect	PLB 574	HUI3	PLB or parents
6	Feeny 2004 [69]	Canada	McMaster	ELBW (≤1000 g); normal BW controls matched by age, sex, and SES	Mean 14	Direct	PLB 140; C 124	HUI2; HUI3	PLB
7	Gray 2007 [78]	UK	ELGA	GA < 29 weeks in mainstream school; normal term controls	15–16	Indirect	PLB 140; C 108	HUI3	PLB
8	Greenough 2004 [79]	UK	RSV infection	Median GA 27 weeks and BW 934 g with chronic lung disease, 17.4% hospitalised for RSV in first two years after birth	5	Indirect	PLB 190	HUI2; HUI3	Parents
9	Greenough 2014 [80]	UK	UKOS	EP (<29 weeks) schoolchildren	11–14	Indirect	PLB 319	HUI3	PLB; parents
10	Hille 2005 [81]	The Netherlands	POPS	VP (<32 weeks) and/or VLBW (<1500 g)	14	Indirect	PLB 853	HUI3	PLB or parents
11	Hille 2007 [82]	The Netherlands	POPS	VP (<32 weeks) and/or VLBW (<1500 g)	19	Indirect	PLB 705	HUI3	PLB or parents
12	Hollanders 2019 [83]	The Netherlands	POPS	VP (<32 weeks) and/or VLBW (<1500 g)	19	Indirect	PLB 705	HUI3	PLB or parents
13	Huhtala 2016 [84]	Finland	PIPARI	VP (<37 weeks) and/or VLBW (≤1500 g); normal term controls from same birth hospital matched by sex	7–8	Indirect	PLB 155; C 129	17D	PLB w/parents
14	Jain 2022 [60]	Ireland, UK	EPICure	EP (<26 weeks); normal term controls who are mainstream schoolmates matched by age, sex, and ethnicity	19	Indirect	PLB 128; C 65	HUI3	PLB
15	James 2003 [61]	Jamaica	Jamaican cohort	LBW (<2500 g); normal BW controls	11–12	Indirect	PLB 96; C 110	HUI2	PLB
16	Liu 2021 [85]	New Zealand	PIANO	VP (<30 weeks) and/or VLBW (<1500 g)	7	Indirect	PLB 127	HUI2	Caregivers
17	Ni 2021 [63]	Ireland, UK	EPICure	EP (<26 weeks); normal term controls recruited at age six years	11, 19 (RM)	Indirect	At age 19: PLB 129; C 65	HUI3	Parents
18	Ni 2022 [86]	UK	EPICure, EPICure2	EP (<26 weeks); normal term controls	11	Indirect	PLB 288; C 261	HUI3	Parents
19	Peart 2021 [67]	Australia	VICS 1991-2, 1997, 2005	EP (<28 weeks) and/or ELBW (<1000 g); normal term/BW controls matched for expected term date of EP/ELBW person, sex, and SES recruited from same birth hospitals	8	Indirect	PLB 475; C 570	HUI2; HUI3	Parents
20	Petrou 2009 [87]	Ireland, UK	EPICure	EP (<26 weeks); normal term controls who are mainstream schoolmates matched by age, sex, and ethnicity	11	Indirect	PLB 190; C 141	HUI3	Parents
21	Petrou 2010 [88]	Ireland, UK	EPICure	EP (<26 weeks); normal term controls who are mainstream schoolmates matched by age, sex, and ethnicity	11	Indirect	PLB 190; C 141	HUI2; HUI3	Parents
22	Petrou 2013 [89]	Ireland, UK	EPICure	EP (<26 weeks); normal term controls who are mainstream schoolmates matched by age, sex, and ethnicity	11	Indirect	PLB 190; C 141	HUI2; HUI3	Parents
23	Quinn 2004 [90]	US	CRYO-ROP	VLBW (≤1250 g) with and without threshold retinopathy of prematurity	10	Indirect	PLB 346	HUI3	Parents or caregivers
24	Rautava 2009 [91]	Finland	PERFECT	VP (<32 weeks) and/or VLBW (≤1500 g); normal term/BW controls born in the same hospital matched by sex	5	Indirect	PLB 588; C 176	17D (modified)	Parents
25	Roberts 2011 [70]	Australia	VICS 1997	EP (<28 weeks) and/or ELBW (<1000 g); normal term/BW controls matched for expected term date of EP/ELBW person, sex, and SES recruited from same birth hospitals	8	Direct	PLB 189; C 173	HUI2	Parents
26	Roberts 2013 [59]	Australia	VICS 1991-2	EP (<28 weeks) and/or ELBW (<1000 g); normal term/BW controls matched for expected term date of EP/ELBW person, sex, and SES recruited from same birth hospitals	18	Indirect	PLB 194; C 148	HUI3	PLB
27	Saigal 1994 [71]	Canada	McMaster	ELBW (≤1000 g); normal BW controls matched by age, sex, and SES	8	Direct	PLB 156; C 145	HUI2	Clinicians
28	Saigal 1994b [92]	Canada	McMaster	ELBW (≤1000 g); normal BW controls matched by age, sex, and SES	8	Indirect	PLB 156; C 145	HUI2	Clinicians
29	Saigal 1996 [93]	Canada	McMaster	ELBW (≤1000 g); normal BW controls matched by age, sex, and SES	Mean 14	Indirect	PLB 141; C 124	HUI2	PLB or parents
30	Saigal 1998 [72]	Canada	McMaster	ELBW (≤1000 g); normal BW controls aged 8 years old	Mean 14	Direct	PLB 141; C 123	HUI2	PLB; parents
31	Saigal 2000 [94]	Canada	McMaster	ELBW (≤1000 g); normal BW controls matched by age, sex, and SES	Mean 14	Indirect	PLB 149; C 126	HUI2	Parents
32	Saigal 2005 [43]	Australia, Canada	CHSCS-PS development	VLBW (<1500 g); normal BW controls; cerebral palsy patients	2.5–5	Direct	PLB 251; C 50	CHSCS-PS	Parents; clinicians
33	Saigal 2006 [95]	Canada	McMaster	ELBW (≤1000 g); normal BW controls matched by age, sex, and SES	Mean 23	Indirect	PLB 143; C 130	HUI2	PLB or parents
34	Saigal 2016 [96]	Canada	McMaster	ELBW (≤1000 g); normal BW controls matched by age, sex, and SES	Mean 14	Indirect	PLB 139; C 124	HUI3	PLB
35	Selman 2023 [65]	Australia	VICS 1991-2	EP (<28 weeks) and/or ELBW (<1000 g); normal term/BW controls matched for expected term date of EP/ELBW person, sex, and SES recruited from same birth hospitals	18, 25 (RM)	Indirect	At age 25: PLB 165; C 131	HUI3	PLB
36	Uusitalo 2020 [97]	Finland	PIPARI	VP (<37 weeks) and/or VLBW (≤1500 g)	11	Indirect	PLB 170	17D	PLB
37	van Dommelen 2014 [98]	The Netherlands	POPS	SGA by weight or length or with a low head circumference or low BW adjusted for length	19	Indirect	PLB 334	HUI3	PLB or parents
38	van Lunenburg 2013 [66]	The Netherlands	POPS	VP (<32 weeks) and/or VLBW (<1500 g)	19, 28 (RM)	Indirect	At age 28: PLB 314	HUI3	PLB
39	Verrips 2001 [73]	The Netherlands	POPS	VP (<32 weeks) and/or VLBW (<1500 g)	14	Direct	PLB 203	HUI3	PLB; parents
40	Verrips 2008 [99]	Canada, Germany, The Netherlands	BLS, McMaster, POPS	ELBW (≤1000 g)	BLS 13, McMaster mean 14, POPS 14	Indirect	PLB 341	HUI3	PLB or caregivers
41	Verrips 2012 [62]	The Netherlands	POPS	VP (<32 weeks) and/or VLBW (<1500 g)	14, 19 (RM)	Indirect	At age 19: PLB 684	HUI3	PLB or parents/caregivers
42	Wolke 2013 [74]	Germany	BLS	VP (<32 weeks) and/or VLBW (<1500 g); normal term/BW controls from same birth hospitals matched by sex and family SES	13	Direct	PLB 294; C 282	HUI3	PLB; parents

^1^ Evidence is ‘direct’ if the study explicitly aimed to assess the psychometric performance of one or more childhood-specific or compatible MAUI; ‘indirect’ if not. ^2^ Only the MAUIs that are specific or compatible with the childhood population are listed. Abbreviation: BLS: Bavarian Longitudinal Study; C: controls; CRYO-ROP: Cryotherapy for Retinopathy of Prematurity study; ELBW: extremely low birthweight; EP: extremely preterm; GA: gestational age; MAUI: multi-attribute utility instrument; PERFECT: Performance, Effectiveness, and Cost of Treatment Episodes Preterm Infant study; PIANO: Protein, Insulin, and Neonatal Outcomes study; PIPARI: the Development and Functioning of Very Low Birth Weight Infants from Infancy to School Age; PLB: preterm and/or low birthweight; POPS: Project on Preterm and Small-for-Gestational-Age Infants; RM: repeat measurements; RSV: respiratory syncytial virus; SES: socioeconomic status; UKOS: United Kingdom Oscillation Study; VICS: Victorian Infant Collaborative Study; VLBW: very low birthweight; VP: very preterm.

**Table 2:** Characteristics of psychometric assessments conducted by included studies.

		n	%
Whether study had a direct aim of assessing psychometric performance	Yes	46	25.8
	No	132	74.2
	Total	178	100.0
PLB population type	EP or ELBW	95	53.4
	VP or VLBW	80	44.9
	LBW	3	1.7
	Total	178	100.0
Target age group	(1) Infants and preschool children aged < 5 years	7	3.9
	(2) Pre-adolescents aged 5–11 years	67	37.6
	(3) Adolescents aged 12–17 years	54	30.3
	(4) Adults aged ≥ 18 years	35	19.7
	(2) and (4)	3	1.7
	(3) and (4)	12	6.7
	Total	178	100.0
Respondent type	Self-report by PLB person	63	35.4
	Self-report by PLB person supported by proxy	33	18.5
	Proxy report only	82	46.1
	Total	178	100.0
Administration mode	Self-administered by a PLB person or proxy	126	70.8
	Interviewer-administered	29	16.3
	Mix of self- and interviewer-administered	15	8.4
	Unclear	8	4.5
	Total	178	100.0

Abbreviation: EP/ELBW: extremely preterm and/or extremely low birthweight; LBW: low birthweight; PLB: preterm and/or low birthweight; VP/VLBW: very preterm and/or very low birthweight.

**Table 3:** Evidence gaps by psychometric property and multi-attribute utility instrument.

N (% of ‘+’)	IC	TR	IR	IM	PC	CV	SV	CCV	KV	HT	CNV	DV	EV	CRV	PV	RE	AC	ITR	Total
All studies (n = 42)																			
17D									4 (50.0)	3 (0.0)							1 (0.0)	3 (0.0)	11
CHSCS-PS		1 (0.0)	1 (0.0)			1 (100)			1 (100)	1 (100)				1 (100)			1 (100)		7
HUI2					1 (100)	1 (0.0)			22 (63.6)	9 (44.4)	1 (0.0)						9 (0.0)	3 (0.0)	46
HUI3	1 (0.0)			3 (0.0)	5 (0.0)				41 (58.5)	20 (55.0)	2 (0.0)					6 (0.0)	17 (5.9)	19 (0.0)	114
Total	1	1	1	3	6	2	0	0	68	33	3	0	0	1	0	6	28	25	178
Studies targeting children (n = 28)																			
17D									4 (50.0)	3 (0.0)							1 (0.0)	3 (0.0)	11
CHSCS-PS		1 (0.0)	1 (0.0)			1 (100)			1 (100)	1 (100)				1 (100)			1 (100)		7
HUI2					1 (100)	1 (0.0)			21 (61.9)	9 (44.4)	1 (0.0)						8 (0.0)	3 (0.0)	44
HUI3				3 (0.0)	5 (0.0)				28 (57.1)	9 (55.6)	1 (0.0)						9 (11.1)	11 (0.0)	66
Total	0	1	1	3	6	2	0	0	54	22	2	0	0	1	0	0	19	17	128
Studies targeting adults or both adults and children (n = 14)																			
HUI2									1 (100)								1 (0.0)		2
HUI3	1 (0.0)								13 (61.5)	11 (54.5)	1 (0.0)					6 (0.0)	8 (0.0)	8 (0.0)	48
Total	1	0	0	0	0	0	0	0	14	11	1	0	0	0	0	6	9	8	50

Psychometric properties: IC: internal consistency; TR: test–retest reliability; IR: inter-rater reliability; IM: inter-modal reliability; PC: proxy–child agreement; CV: content validity; SV: structural validity; CCV: cross-cultural validity; KV: known-group validity; HT: hypothesis testing; CNV: convergent validity; DV: discriminant validity; EV: empirical validity; CRV: concurrent validity; PV: predictive validity; RE: responsiveness; AC: acceptability; ITR: interpretability. The number of criteria rating outputs is shown in the cells; the parenthesis contains the percentage of outputs that is ‘+’ for the psychometric property and instrument.

### 3.5. Psychometric Assessment Methods and Performance by Property

This section addresses the third review objective and describes the psychometric assessment methods and performance of MAUIs for the properties with at least one criteria rating output. Figure S3 in the Supplementary Information shows the frequency of the criteria rating outputs as absolute numbers and proportions by instrument and psychometric property.

#### 3.5.1. Internal Consistency

Only one criteria rating output was available for internal consistency. Verrips and colleagues [62] estimated the correlations between the change in the HUI3 utility score of VP/VLBW individuals from ages 14 to 19 and the changes in the HUI3 dimension-specific single-attribute utility scores. This was interpreted as an assessment of item–total correlations. However, without an estimate of Cronbach’s alpha, no judgement could be reached on internal consistency, and the criteria rating output was ‘?’.

#### 3.5.2. Test–Retest Reliability

The only assessment of test–retest reliability was conducted by Saigal and colleagues [43] for the CHSCS-PS. Parental responses were obtained 14 days apart and assessed for agreement. The percentage agreements for each dimension were high, ranging between 86% and 100%. However, the Kappa statistics for agreement were generally low, with five of the seven dimensions for which Kappa values were calculated having values below 0.70. Hence, the criteria rating output was ‘±’.

#### 3.5.3. Inter-Rater Reliability

The CHSCS-PS was again the only MAUI with inter-rater reliability evidence concerning the level of agreement between parental and clinician responses [43]. Percentage agreements were high (>80%) for objective dimensions such as mobility and lower (72–80%) for more subjective dimensions, including self-care and behaviour. Kappa statistics ranged widely between 0.30 and 1.00, resulting in an output of ‘±’.

#### 3.5.4. Inter-Modal Reliability

Verrips and colleagues [73] provided the only inter-modal reliability evidence concerning the agreement between self- and interviewer-administered HUI3 responses. Levels of agreement were consistently low (output ‘−’) for dimension-level responses from children. The Kappa statistics were below 0.70 for all dimensions regarding the agreement between mail and telephone interviews and for all but one dimension regarding the agreement between mail and face-to-face interviews. For parent responses, the statistics were below 0.70 for all but one dimension regarding both sets of agreement. There were, moreover, statistically significant (*p* < 0.05) differences in the mean HUI3 utility score and in the mean HUI3 unweighted sum of dimension levels between mail and interviewer administrations.

#### 3.5.5. Proxy–Child Agreement

Saigal and colleagues [72] found high percentage agreements (80–100%) between the HUI2 dimension responses given by ELBW and normal birthweight children and their parents (output ‘+’). Evidence from two studies [73,74] suggested that proxy–child agreement was mixed for the HUI3. Verrips and colleagues [73] found no statistically significant differences in mean HUI3 utility score and mean HUI3 unweighted sum between interview-administered parental and child responses but significant differences between self-administered responses (output ‘±’). The Kappa statistics for agreements between dimension responses were consistently low (output ‘−’). Wolke and colleagues [74] found statistically a significant difference in mean HUI3 utility score between parental and child responses (output ‘−’); at the dimension level, percentage agreements were above 70%; however, the Kappa statistics were below 0.70 for most dimensions (output ‘±’).

#### 3.5.6. Content Validity

Content validity evidence was available for the HUI2 and CHSCS-PS. Saigal and colleagues (1994) [71] perceived that the HUI2, in its original form, is not suitable for ELBW children. Thus, based on a literature review and their experiences, the authors added two dimensions, namely, behaviour and general health, which were subsequently piloted and validated in a prospective application [71]. The need for additional dimensions indirectly suggests that the content validity of the HUI2 for the PLB population is low. Almost all studies that applied the HUI2 also excluded its fertility dimension. Though not given a criteria rating output, this again indicates the low content validity of the HUI2. The lack of evidence for the content validity of the HUI3 precludes judgment on whether the most frequently applied MAUI for the PLB population adequately captures the health constructs of relevance.

By contrast, Saigal and colleagues (2005) [43] provided direct evidence of the content validity of the CHSCS-PS. The conceptual framework and ten dimensions of the CHSCS-PS were drawn from the HUI2/3 and the additional two dimensions (behaviour and general health) were identified by a literature review. Age-appropriate response levels were identified from standardised tests and paediatric experts. Piloting was conducted before producing the draft version, which was then applied to 80 children, 18 parents, and three paediatricians for a consensus exercise. Neonatologists and paediatricians who applied the draft version provided structured and qualitative feedback. The larger-scale prospective application was conducted in two samples of VLBW children and a sample of cerebral palsy patients. Therefore, although PLB children were involved only in the last phase of its development, the CHSCS-PS has the highest likelihood of measuring the HRQoL constructs relevant to the PLB population.

#### 3.5.7. Known-Group Validity

A large proportion (38.2%) of identified psychometric evidence concerned known-group validity. When comparing the utility scores and/or dimension-level responses between the PLB group and full-term/normal birthweight controls, studies that stated their a priori hypothesis generally expected to find significantly lower HRQoL for the PLB group [43,60,61,67,70,74,78,84,95,96,97]. The sole exception was the study by Roberts and colleagues (2013) [59], which expected similar HRQoL between controls and EP/ELBW adults born in Australia prior to the introduction of surfactants. Twelve studies did not clearly state their a priori hypothesis and were assumed to have expected lower HRQoL for the PLB group relative to controls [64,68,69,71,75,76,82,83,87,92,93,94]. Several studies tested hypotheses on subgroup differences in HRQoL within the PLB population [77,79,80,81,85,86,88,89,90,96,99]: e.g., between extremely preterm subgroups with and without a neurodevelopmental disability [89].

Hypothesised subgroup differences were found (output ‘+’) in 50% of known-group validity assessments for the 17D, 100% for the CHSCS-PS, 63.6% for the HUI2, and 58.5% for the HUI3. However, the numbers of assessments were smaller for the 17D (n = 4) and CHSCS-PS (n = 1) than for the HUI2 (n = 22) and HUI3 (n = 41), precluding any straightforward judgement on the best performing MAUI.

#### 3.5.8. Hypothesis Testing

Evidence on hypothesis testing mostly comprised results of multivariate regression analyses. For example, Selman and colleagues [65] hypothesised that HRQoL would be lower for EP/ELBW adults than full-term/normal birthweight controls at ages 18 and 25, adjusted for maternal education and social class. They conducted quantile regressions and logistic regressions with the median HUI3 utility score and the presence of any deficit in each HUI3 dimension, respectively, as the dependent variable. The hypotheses were met, and, thus, the two assessments (one each for utility score and dimension response) received a ‘−’ output. Overall, 0% of the hypothesis-testing assessments received ‘+’ for the 17D (out of n = 3), 100% for the CHSCS-PS (n = 1), 44.4% for the HUI2 (n = 9), and 55% for the HUI3 (n = 20).

#### 3.5.9. Convergent Validity

Three criteria rating outputs were available for convergent validity, one for the HUI2 and two for the HUI3. Feeny and colleagues [69] assessed the agreement between standard gamble utility and HUI2 and HUI3 utility scores. The standard gamble utility and HUI2 utility scores were found to be comparable at the group level, with a lack of a statistically or clinically significant difference between their means. However, their agreement at the individual level was low with an intraclass correlation coefficient of 0.15 (output ‘±’). Agreements between the standard gamble utility and HUI3 utility scores were low at both group and individual levels (output ‘−’). Bolbocean and colleagues [68] found low agreement between HUI3 and SF-6D utility scores at group and individual levels (output ‘−’).

#### 3.5.10. Concurrent Validity

Saigal and colleagues (2005) [43] conducted the only assessment for concurrent validity. They hypothesised statistically significant negative associations between CHSCS-PS dimension levels (higher levels indicating worse HRQoL) and the following standardised and well-known measures of disability, such as between the Bayley Scales of Infant Development II Revised Psychomotor Development Index and the mobility and the dexterity dimensions of CHSCS-PS. Hypothesised associations were found in each case (output ‘+’).

#### 3.5.11. Responsiveness

No study conducted a longitudinal assessment of the effectiveness of a specific healthcare intervention targeting the PLB population. All six criteria rating outputs for responsiveness, therefore, concerned the natural history of the PLB population’s HRQoL measured by the HUI3. Ni and colleagues (2021) [63] found statistically and clinically significant declines in mean HUI3 utility scores from ages 11 to 19 for both extremely preterm and full-term groups in the EPICure cohort. Verrips and colleagues (2012) [62] likewise found a non-significant decline in the mean HUI3 utility score for a VP/VLBW cohort between the ages of 14 and 19. By contrast, van Lunenburg and colleagues [66] found a statistically significant increase in mean HUI3 utility scores from ages 19 to 28 for the same cohort. However, none of the studies stated their a priori hypothesis or included a reference measure to help judge whether the (lack of) change in HUI3 measures a (lack of) change in the HRQoL construct. Hence, their outputs were ‘?’.

#### 3.5.12. Acceptability

Twelve studies assessed acceptability but only concerning one criterion (e.g., missing data rate), which meant that their assessments (n = 15) received the output ‘?’ [59,61,67,68,69,75,78,81,82,83,96,97]. Another 12 studies assessed multiple criteria [43,62,64,71,72,77,86,90,93,94,95,99]. The 17D had only one assessment showing low missing data [97]. The CHSCS-PS was assessed to have low levels of missing data, a short response time, and a high number of unique health states [43]. Of the studies that assessed multiple criteria, the HUI2 consistently showed a high ceiling effect [71,72,93,94,95]. The HUI3 likewise showed a high ceiling effect [62,64,77,86,90,99], and a mix of high [64,86] and low [77,90] missing data rates. Also worth noting is that around a quarter of the studies (n = 10) employed proxies (e.g., parents) to assist in the self-response from the severely impaired subgroups within their PLB samples: the 17D applied in PLB children [84]; the HUI2 in PLB children [93] and adults [95]; and the HUI3 in PLB children [81,99], adults [77,82,83,98], and both [62]. This suggests that the acceptability of these MAUIs for severely impaired PLB persons is low.

#### 3.5.13. Interpretability

Evidence on interpretability mainly consisted of using the minimal clinically important difference (MID) sourced from the literature. For the HUI2 and HUI3, a change or difference of 0.03 in utility score and 0.05 in single-attribute utility score were cited to be the MID [100,101]. For the 17D, a difference of 0.03 in utility score was likewise cited based on the MID for the adult-specific 15D [102]. Two studies made external comparisons to help interpret the HRQoL of their respective PLB samples. Hille and colleagues (2007) [82] concluded that the HUI3 utility scores of VP/VLBW adults in their sample were similar to those of the general population. Uusitalo and colleagues [97] concluded that the 17D utility scores and dimension scores of VP/VLBW children indicated *higher* HRQoL than that observed in the general childhood population. No study derived the population norm or MID for HRQoL de novo; hence, none received an output of ‘+’.

## 4. Discussion

This study is the first review of the psychometric performance of childhood-specific or compatible MAUIs in the PLB population. The psychometric evidence base developed from the 42 included studies should facilitate the selection of scientifically rigorous MAUI(s) for clinical research and health economic evaluations in this population, as well as motivate further psychometric research to fill the identified gaps in the current evidence base. The review also summarised the psychometric assessment methods and performance of the four MAUIs applied in this population (17D, CHSCS-PS, HUI2, and HUI3) by psychometric property. No MAUI consistently outperformed the others across all properties for which evidence was available. This suggests that selection should depend on which properties are most relevant for the research and clinical practice setting in which the MAUI is applied. The CHSCS-PS had the greatest number of properties for which there was at least one ‘+’ output but had the lowest number of outputs from a single study and targeted a narrow age group of 2–4 years. The HUI3 was the most commonly applied childhood-compatible MAUI in PLB populations but had mixed psychometric performance across the properties and lacked any evidence for content validity.

Other major gaps in the psychometric evidence base were identified for this population. First, the range of psychometric properties covered was narrow: five properties (structural validity, cross-cultural validity, discriminant validity, empirical validity, and predictive validity) lacked any evidence, and another seven (internal consistency, test–retest reliability, inter-rater reliability, inter-modal reliability, convergent validity, responsiveness, and interpretability) lacked any positive rating of evidence. The review revealed that known-group validity was the property with the greatest psychometric evidence, reflected in 38.2% of assessments. Evidence on reliability (i.e., internal consistency and test–retest, inter-rater, and inter-modal reliabilities) and proxy–child agreement were particularly lacking, with only 12 outputs in total, representing just 6.7% of the 178 outputs. In comparison, for all childhood populations as identified in the previous psychometric review [35], evidence for these five properties comprised 15.1% of all outputs.

Second, the range of MAUIs covered was similarly narrow, comprising only four. Moreover, the evidence volume was skewed towards the HUI system, with the HUI3 being applied in 64.0% of the identified assessments and the HUI2/3 in 89.9%. In comparison, the respective proportions of assessments by these measures were 28.9% and 50.9% for all childhood populations [35]. The frequent use of the HUI2/3 in the PLB population has the strength to make the HRQoL results comparable across different cohorts and studies. In addition, a key strength of the HUI2/3 is their applicability in both children (as young as five years old if a proxy report is used) and adults [25], making it possible to assess the HRQoL transitions from childhood into adulthood [65]. That said, the lack of psychometric evidence for other MAUIs—including those that are also members of a family of measures applicable across both children and adults (e.g., EQ-5D-Y and EQ-5D; 17D, 16D, and 15D; AQoL-6D adolescent and adult versions)—makes it difficult to judge whether the HUI2/3 really are the best measurement options for assessing HRQoL in PLB populations. This is particularly so considering that the HUI2 appears to lack content validity for PLB children [71], while the HUI3 lacks any evidence of content validity. The relatively frequent need to rely on proxy support to obtain responses from PLB children and adults again suggests that the HUI2/3 may not be best suited for this population, at least for obtaining self-reported responses from its more disabled members [62,77,81,82,83,93,95,98,99].

Content validity is particularly important for the measurement of HRQoL in PLB populations, which typically adapt to disabilities such that their self-reported levels on the ‘subjective’ dimensions of HRQoL (e.g., socio-emotional functioning) are broadly comparable to those of their full term/normal birthweight peers, this phenomenon being labelled the ‘disability paradox’ [95]. The subjective dimensions subsequently correlate poorly with health status measures or with more ‘objective’ or observable dimensions of HRQoL such as physical functioning [95]. The key issue, then, is the relative importance of the different dimensions for the PLB population’s HRQoL, and MAUIs that place different relative emphases (via relative numbers of dimensions or items or the preference weights placed on health states through their value sets) may struggle to capture the level and change in the PLB population’s HRQoL. Content validation aims to verify whether the relative emphasis placed by a given PROM is acceptable to the target population [44]. Post-development content validation is also possible, whereby surveys and qualitative studies evaluate the instruments’ conceptual relevance to the target population’s HRQoL constructs of importance [103,104]. The lack of content validity evidence for HUI3 becomes more problematic in this context.

A key strength of this study is its focus on the psychometric performance of MAUIs in a specific population. The previous psychometric review covering all childhood populations provided top-line evidence only [35], while the current review disentangles the evidence for PLB populations regardless of age. Policymakers engaged in health technology appraisal or health needs assessment for PLB populations can check the current catalogue of psychometric evidence to verify whether credible policy directions could be inferred from primary studies that apply a given MAUI to a PLB sample. For instance, a health technology appraisal agency could receive the results of an economic evaluation study that used the EQ-5D-Y to measure the health utility impact of an intervention targeting a childhood PLB sample. The agency should then be cautious in drawing any firm policy conclusions given that no psychometric evidence currently exists concerning the application of the EQ-5D-Y in PLB populations. Investigators designing research to inform such agencies should also be cautious in applying the EQ-5D-Y. The catalogue should likewise be useful for the research community, not only in identifying the psychometric evidence gaps specific to PLB populations but also in detailing the prevalent methodological issues. One such issue is the specification of an a priori hypothesis before the HRQoL comparison between PLB groups and controls. Due to the disability paradox, health status measures and neonatal factors are often poor guides for setting the hypothesis, and it may be that the research community should seek a consensus on the appropriate ways of doing so.

This study nevertheless has several limitations. First, although the coverage of adult PLB populations was a strength, the non-coverage of adult-specific MAUIs such as the SF-6D in adult PLB populations curtailed the range of psychometric evidence. Second, the assumption that the studies that did not state their a priori hypothesis were expecting a lower HRQoL for the PLB group than controls may have underestimated the psychometric performance of the MAUIs: a lack of significant HRQoL difference was interpreted as poor psychometric performance when it could have accurately reflected comparable HRQoL between the two groups [64]. That said, the assumption was applied equally for all MAUIs such that the evaluation of relative performance would be little affected. Third, it is possible that the database selection introduced bias in the study inclusion, even though the search strategy had been designed and implemented by an information specialist. For example, the inclusion of the Cochrane Central Register of Controlled Trials may have improved the identification of RCTs in PLB populations and thus evidence on responsiveness. Finally, although the psychometric criteria used for the evaluation (see Table S8 in the Supplementary Information) were informed by several guidelines, it is possible that some criteria were missed. The criteria for modern psychometric theories (e.g., Rasch analysis) were also omitted. That said, a strength of this review is that it conducted case-by-case judgements of psychometric performance with the methods and results detailed in the online Excel catalogue. This mitigates the risk that certain criteria affecting the measurement performance of MAUIs were neglected by the review.

This review points to several avenues of further research, most importantly those addressing the identified psychometric evidence gaps. First, there is a significant paucity of evidence from low- and middle-income countries. Second, there is a particular need for empirical validity evidence concerning the degree to which the MAUI utility values reflect people’s preferences over health, often measured by self-reported health status [32,105]. Given the disability paradox and the resulting emphasis on the subjective dimensions of HRQoL by the PLB population, empirical validity may provide a more accurate picture of a given MAUI’s construct validity than known-group validity. Third, there is a strong need for more evidence on proxy–child agreement. Across all childhood conditions, proxy–child agreement has been shown to be lower for subjective dimensions of HRQoL than for its observable dimensions [26]. Therefore, the importance of subjective dimensions of HRQoL in PLB children likely means that proxy–child agreement is low and more evidence is needed. Fourth, there is a large scope for applying and validating further childhood-specific or compatible MAUIs in this population. The CHSCS-PS had the highest number of psychometric properties with at least one ‘+’ rating; however, its evidence came from a single study [43]. Further application of the CHSCS-PS is thus warranted, as well as the development of its first value set for health utility derivation. Finally, only five studies made use of the longitudinal dimension of the PLB cohorts [62,63,64,65,66]; more evidence on responsiveness could be obtained through further longitudinal analyses.

## 5. Conclusions

This systematic review provides comprehensive and up-to-date evidence on the psychometric performance of generic childhood-specific or compatible MAUIs that have been applied in preterm and/or low birthweight populations. The catalogue of evaluated psychometric evidence provides a valuable resource for researchers and policymakers—particularly those involved in cost-effectiveness analysis, modelling, and decision-making—in selecting MAUI(s) for applications targeting this population as well as in interpreting the results of studies that applied the MAUI(s). No MAUI consistently outperformed others across all properties, meaning that selection would depend on which properties are most relevant for further application. Important psychometric evidence gaps were identified, which should motivate further psychometric research, such as the paucity of evidence around reliability and proxy–child agreement and the lack of evidence on content validity for the HUI3. The commonly observed issues in psychometric assessment design and methods, such as the clear statement of the a priori hypothesis for testing associations and changes, should likewise inform future psychometric studies.

## Data Availability

The Excel file containing the individual criteria rating outputs and the rationale is available online.

## Supplementary Materials

The following supporting information can be downloaded at: https://www.mdpi.com/article/10.3390/children10111798/s1, Supplementary Information contains the PRISMA checklist, Tables S1–S10, and Figures S1–S3. The Excel file containing the individual criteria rating outputs and the rationale is available online.

## Abbreviations

16D	16-dimensional health-related measure
17D	17-dimensional health-related measure
AHUM	Adolescent Health Utility Measure
AQoL-6D Adolescent	Assessment of Quality of Life, 6-Dimensional, Adolescent
CH-6D	Child Health—6 Dimensions
CHSCS-PS	Comprehensive Health Status Classification System—Preschool
CHU9D	Child Health Utility—9 Dimensions
EP/ELBW	extremely preterm and/or extremely low birthweight
EQ-5D-Y-3L	EuroQoL 5 Dimensional questionnaire for Youth 3 Levels
EQ-5D-Y-5L	EuroQoL 5 Dimensional questionnaire for Youth 5 Levels
EQ-TIPS	EuroQoL Toddler and Infant Populations
HRQoL	health-related quality of life
HUI2	Health Utilities Index 2
HUI3	Health Utilities Index 3
IQI	Infant health-related Quality of life Instrument
MAUI	multi-attribute utility instrument
PLB	preterm and/or low birthweight
PRISMA	preferred reporting items for systematic reviews and meta-analyses
PROM	patient-reported outcome measure
QWB	Quality of Well-Being scale
RCT	randomised controlled trial
TANDI	Toddler and Infant health related quality of life instrument
VP/VLBW	very preterm and/or very low birthweight
