# Supplementary material for: Psychometric Performance of Generic Childhood Multi-Attribute Utility Instruments in Preterm and Low Birthweight Populations: A Systematic Review
Source: Children (Basel). 2023 Nov 10;10(11):1798. doi: 10.3390/children10111798 (PMC10670192; doi:10.3390/children10111798)
Supplement: Supplementary file 1 [file children-10-01798-s001.zip › children-2666032-supplementary.pdf]

Psychometric Performance of Generic Childhood Multi-Attribute Utility Instruments in Preterm and Low Birthweight Populations

Supplementary Information

Joseph Kwon [1], Corneliu Bolbocean [1], Olu Onyimadu [1], Nia Roberts [2] and Stavros Petrou [1,*]

[1] Nuffield Department of Primary Care Health Sciences, University of Oxford, Oxford OX2 6GG, UK; joseph.kwon@phc.ox.ac.uk (J.K.); corneliu.bolbocean@phc.ox.ac.uk (C.B.); olu.onyimadu@phc.ox.ac.uk (O.O.)
2 Bodleian Health Care Libraries, University of Oxford, Oxford OX3 9DU, UK; nia.roberts@bodleian.ox.ac.uk
* Correspondence: stavros.petrou@phc.ox.ac.uk
.

PRISMA 2020 checklist

Section and Topic	Item #	Checklist item	Location where item is reported
**TITLE**			
Title	1	Identify the report as a systematic review.	Title
**ABSTRACT**			
Abstract	2	See the PRISMA 2020 for Abstracts checklist.	Word count constraint meant not all checklist items met
**INTRODUCTION**			
Rationale	3	Describe the rationale for the review in the context of existing knowledge.	'1. Background'
Objectives	4	Provide an explicit statement of the objective(s) or question(s) the review addresses.	'1. Background' final paragraph
**METHODS**			
Eligibility criteria	5	Specify the inclusion and exclusion criteria for the review and how studies were grouped for the syntheses.	'2.1 Data sources and study selection'
Information sources	6	Specify all databases, registers, websites, organisations, reference lists and other sources searched or consulted to identify studies. Specify the date when each source was last searched or consulted.	'2.1 Data sources and study selection'
Search strategy	7	Present the full search strategies for all databases, registers and websites, including any filters and limits used.	Tables S1-S7
Selection process	8	Specify the methods used to decide whether a study met the inclusion criteria of the review, including how many reviewers screened each record and each report retrieved, whether they worked independently, and if applicable, details of automation tools used in the process.	'2.1 Data sources and study selection'
Data collection process	9	Specify the methods used to collect data from reports, including how many reviewers collected data from each report, whether they worked independently, any processes for obtaining or confirming data from study investigators, and if applicable, details of automation tools used in the process.	'2.2 Data extraction'
Data items	10a	List and define all outcomes for which data were sought. Specify whether all results that were compatible with each outcome domain in each study were sought (e.g. for all measures, time points, analyses), and if not, the methods used to decide which results to collect.	'2.2 Data extraction'
	10b	List and define all other variables for which data were sought (e.g. participant and intervention characteristics, funding sources). Describe any assumptions made about any missing or unclear information.	'2.2 Data extraction'
Study risk of bias assessment	11	Specify the methods used to assess risk of bias in the included studies, including details of the tool(s) used, how many reviewers assessed each study and whether they worked independently, and if applicable, details of automation tools used in the process.	'2.3 Evaluation and data synthesis'
Effect measures	12	Specify for each outcome the effect measure(s) (e.g. risk ratio, mean difference) used in the synthesis or presentation of results.	'2.3 Evaluation and data synthesis'
Synthesis	13a	Describe the processes used to decide which studies were eligible for each synthesis (e.g. tabulating the study	'2.3 Evaluation and data

Section and Topic	Item #	Checklist item	Location where item is reported
methods		intervention characteristics and comparing against the planned groups for each synthesis (item #5)).	synthesis’
	13b	Describe any methods required to prepare the data for presentation or synthesis, such as handling of missing summary statistics, or data conversions.	‘2.3 Evaluation and data synthesis’
	13c	Describe any methods used to tabulate or visually display results of individual studies and syntheses.	‘2.3 Evaluation and data synthesis’
	13d	Describe any methods used to synthesize results and provide a rationale for the choice(s). If meta-analysis was performed, describe the model(s), method(s) to identify the presence and extent of statistical heterogeneity, and software package(s) used.	‘2.3 Evaluation and data synthesis’
	13e	Describe any methods used to explore possible causes of heterogeneity among study results (e.g. subgroup analysis, meta-regression).	‘2.3 Evaluation and data synthesis’
	13f	Describe any sensitivity analyses conducted to assess robustness of the synthesized results.	‘2.3 Evaluation and data synthesis’
Reporting bias assessment	14	Describe any methods used to assess risk of bias due to missing results in a synthesis (arising from reporting biases).	‘2.3 Evaluation and data synthesis’
Certainty assessment	15	Describe any methods used to assess certainty (or confidence) in the body of evidence for an outcome.	‘2.3 Evaluation and data synthesis’
**RESULTS**			
Study selection	16a	Describe the results of the search and selection process, from the number of records identified in the search to the number of studies included in the review, ideally using a flow diagram.	Figure 1
	16b	Cite studies that might appear to meet the inclusion criteria, but which were excluded, and explain why they were excluded.	Table S8
Study characteristics	17	Cite each included study and present its characteristics.	Table 2
Risk of bias in studies	18	Present assessments of risk of bias for each included study.	Table S9 for ‘?’ outcome
Results of individual studies	19	For all outcomes, present, for each study: (a) summary statistics for each group (where appropriate) and (b) an effect estimate and its precision (e.g. confidence/credible interval), ideally using structured tables or plots.	Table S9
Results of syntheses	20a	For each synthesis, briefly summarise the characteristics and risk of bias among contributing studies.	‘3.5 Psychometric assessment methods and performance by property’
	20b	Present results of all statistical syntheses conducted. If meta-analysis was done, present for each the summary estimate and its precision (e.g. confidence/credible interval) and measures of statistical heterogeneity. If comparing groups, describe the direction of the effect.	No statistical synthesis
	20c	Present results of all investigations of possible causes of heterogeneity among study results.	Not relevant

Section and Topic	Item #	Checklist item	Location where item is reported
	20d	Present results of all sensitivity analyses conducted to assess the robustness of the synthesized results.	Not relevant
Reporting biases	21	Present assessments of risk of bias due to missing results (arising from reporting biases) for each synthesis assessed.	Table S9 for '?' outcome
Certainty of evidence	22	Present assessments of certainty (or confidence) in the body of evidence for each outcome assessed.	Table S9 for '?' outcome
**DISCUSSION**			
Discussion	23a	Provide a general interpretation of the results in the context of other evidence.	'4. Discussion'
	23b	Discuss any limitations of the evidence included in the review.	'4. Discussion' 8th paragraph
	23c	Discuss any limitations of the review processes used.	'4. Discussion' 8th paragraph
	23d	Discuss implications of the results for practice, policy, and future research.	'5. Conclusion'
**OTHER INFORMATION**			
Registration and protocol	24a	Provide registration information for the review, including register name and registration number, or state that the review was not registered.	'2. Methods'
	24b	Indicate where the review protocol can be accessed, or state that a protocol was not prepared.	'2. Methods'
	24c	Describe and explain any amendments to information provided at registration or in the protocol.	Not relevant
Support	25	Describe sources of financial or non-financial support for the review, and the role of the funders or sponsors in the review.	'Declarations'
Competing interests	26	Declare any competing interests of review authors.	'Declarations'
Availability of data, code and other materials	27	Report which of the following are publicly available and where they can be found: template data collection forms; data extracted from included studies; data used for all analyses; analytic code; any other materials used in the review.	'Supplementary Information'

Systematic review process, terminology, and objectives

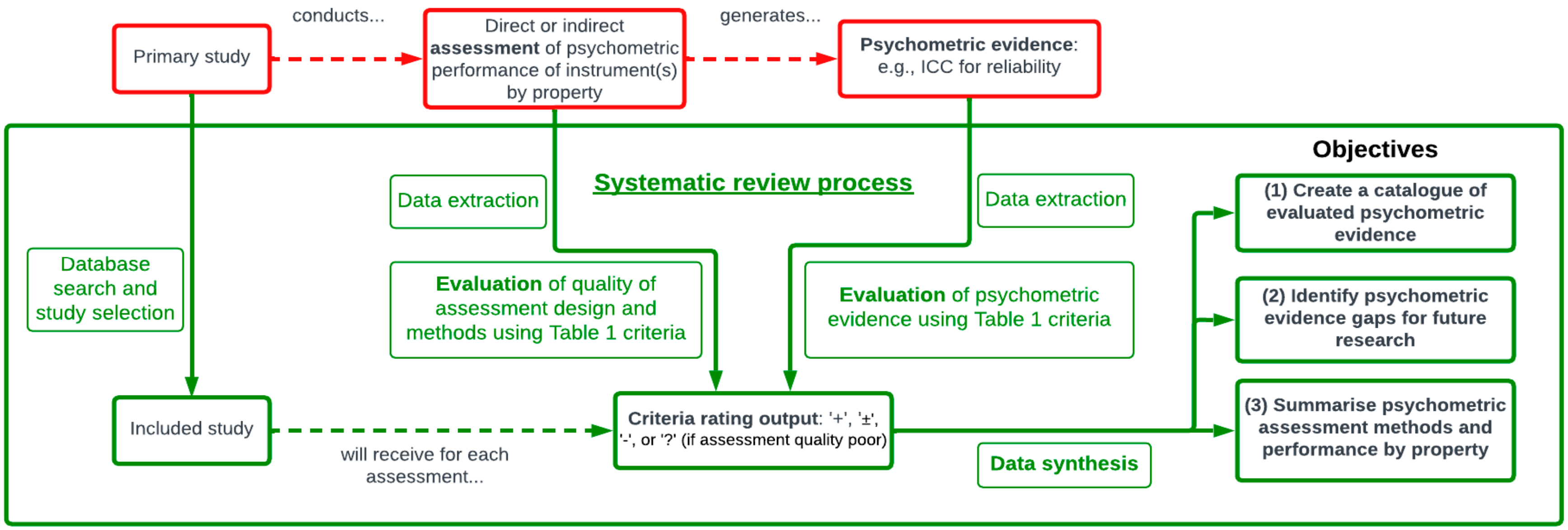

Figure S1. Illustration of the systematic review process, terminology, and objectives. **Abbreviation:** ICC: intraclass correlation coefficient.

Search strategies

Table S1 Medline (Ovid MEDLINE® Epub Ahead of Print, In-Process & Other Non-Indexed Citations, Ovid MEDLINE® Daily and Ovid MEDLINE®) 1946 to 26th April 2023

1	health utilities index.ti,ab,kw.
2	hui mark 2.ti,ab,kw.
3	hui2.ti,ab,kw.
4	hui-2.ti,ab,kw.
5	huiii.ti,ab,kw.
6	hui-ii.ti,ab,kw.
7	hui mark 3.ti,ab,kw.
8	hui3.ti,ab,kw.
9	hui-3.ti,ab,kw.
10	huiiii.ti,ab,kw.
11	hui-iii.ti,ab,kw.
12	1 or 2 or 3 or 4 or 5 or 6 or 7 or 8 or 9
13	child health utility 9d.ti,ab,kw.
14	child health utility 9-d.ti,ab,kw.
15	chu9d.ti,ab,kw.
16	chu-9d.ti,ab,kw.
17	child health utility 9-dimension*.ti,ab,kw.
18	child health utility 9dimension*.ti,ab,kw.
19	13 or 14 or 15 or 16 or 17 or 18
20	assessment of quality of life 6-dimension*.ti,ab,kw.
21	assessment of quality of life 6dimension*.ti,ab,kw.
22	assessment of quality of life 6-d.ti,ab,kw.
23	assessment of quality of life 6d.ti,ab,kw.
24	assessment of qol 6-dimension*.ti,ab,kw.
25	assessment of qol 6dimension*.ti,ab,kw.
26	assessment of qol 6-d.ti,ab,kw.
27	assessment of qol 6d.ti,ab,kw.
28	aqol-6d.ti,ab,kw.
29	aqol6d.ti,ab,kw.
30	20 or 21 or 22 or 23 or 24 or 25 or 26 or 27 or 28 or 29
31	16-dimension*.ti,ab,kw.
32	16dimension*.ti,ab,kw.
33	16-d.ti,ab,kw.
34	16d.ti,ab,kw.
35	31 or 32 or 33 or 34
36	("quality of life" or qol or hrqol or hr-qol or utility or utilities or preference*).ti,ab,kw.
37	35 and 36
38	17-dimension*.ti,ab,kw.
39	17dimension*.ti,ab,kw.
40	17-d.ti,ab,kw.
41	17d.ti,ab,kw.
42	38 or 39 or 40 or 41
43	("quality of life" or qol or hrqol or hr-qol or utility or utilities or preference*).ti,ab,kw.
44	42 and 43
45	ahum.ti,ab,kw.
46	a-hum.ti,ab,kw.
47	adolescent health utility measure*.ti,ab,kw.
48	adolescent hum.ti,ab,kw.
49	45 or 46 or 47 or 48
50	eq-5d-y*.ti,ab,kw.
51	eq-5dy*.ti,ab,kw.
52	eq-5d-5l-y.ti,ab,kw.
53	eq-5d5ly.ti,ab,kw.
54	european quality of life 5d youth.ti,ab,kw.
55	european quality of life 5-d youth.ti,ab,kw.
56	european quality of life 5 dimension* youth.ti,ab,kw.

57	euroqol 5dy.ti,ab,kw.
58	euroqol 5d-y.ti,ab,kw.
59	euroqol 5 dimension* youth*.ti,ab,kw.
60	euroqol 5d youth*.ti,ab,kw.
61	euroqol 5-d youth.ti,ab,kw.
62	50 or 51 or 54 or 55 or 56 or 57 or 58 or 59 or 60 or 61
63	iqi.ti,ab,kw.
64	infant health related quality of life instrument*.ti,ab,kw.
65	infant quality of life instrument.ti,ab,kw.
66	infant qol instrument.ti,ab,kw.
67	infant hrqol instrument*.ti,ab,kw.
68	infant hr-qol instrument.ti,ab,kw.
69	tandi.ti,ab,kw.
70	("toddler and infant" adj2 "health related quality of life").ti,ab,kw.
71	("toddler and infant" adj2 hrqol).ti,ab,kw.
72	("toddler and infant" adj2 hr-qol).ti,ab,kw.
73	("Toddler and Infant" adj3 "european quality of life").ti,ab,kw.
74	("Toddler and Infant" adj3 euroqol).ti,ab,kw.
75	(eq-tips or eqtips).ti,ab,kw.
76	63 or 64 or 65 or 66 or 67 or 68 or 69 or 70 or 71 or 72 or 73 or 74 or 75
77	chscs-ps.ti,ab,kw.
78	comprehensive health status classification system for preschool children.ti,ab,kw.
79	comprehensive health status classification system for pre-school children.ti,ab,kw.
80	77 or 78 or 79
81	ch-6d.ti,ab,kw.
82	ch6d.ti,ab,kw.
83	ch 6dimension*.ti,ab,kw.
84	ch 6 dimension*.ti,ab,kw.
85	child health 6d*.ti,ab,kw.
86	child health 6-d*.ti,ab,kw.
87	81 or 82 or 83 or 84 or 85 or 86
88	quality of wellbeing.ti,ab,kw.
89	quality of well-being.ti,ab,kw.
90	qwb.ti,ab,kw.
91	88 or 89 or 90
92	12 or 19 or 30 or 37 or 44 or 49 or 62 or 76 or 80 or 87 or 91
93	exp infant, low birth weight/ or exp infant, premature/
94	(preterm or pre-term or prematur*).ti,ab,kf.
95	("small for gestational age" or sga or (birthweight or birth weight or lbw* or vlbw* or elbw*)).ti,ab,kf.
96	93 or 94 or 95
97	92 and 96

Table S2 Embase (OvidSP) 1974 to 26th April 2023

1	health utilities index.ti,ab,kw.
2	hui mark 2.ti,ab,kw.
3	hui2.ti,ab,kw.
4	hui-2.ti,ab,kw.
5	huiii.ti,ab,kw.
6	hui-ii.ti,ab,kw.
7	hui mark 3.ti,ab,kw.
8	hui3.ti,ab,kw.
9	hui-3.ti,ab,kw.
10	huiiii.ti,ab,kw.
11	hui-iii.ti,ab,kw.
12	1 or 2 or 3 or 4 or 5 or 6 or 7 or 8 or 9
13	child health utility 9d.ti,ab,kw.
14	child health utility 9-d.ti,ab,kw.
15	chu9d.ti,ab,kw.

16	chu-9d.ti,ab,kw.
17	child health utility 9-dimension*.ti,ab,kw.
18	child health utility 9dimension*.ti,ab,kw.
19	13 or 14 or 15 or 16 or 17 or 18
20	assessment of quality of life 6-dimension*.ti,ab,kw.
21	assessment of quality of life 6dimension*.ti,ab,kw.
22	assessment of quality of life 6-d.ti,ab,kw.
23	assessment of quality of life 6d.ti,ab,kw.
24	assessment of qol 6-dimension*.ti,ab,kw.
25	assessment of qol 6dimension*.ti,ab,kw.
26	assessment of qol 6-d.ti,ab,kw.
27	assessment of qol 6d.ti,ab,kw.
28	aqol-6d.ti,ab,kw.
29	aqol6d.ti,ab,kw.
30	20 or 21 or 22 or 23 or 24 or 25 or 26 or 27 or 28 or 29
31	16-dimension*.ti,ab,kw.
32	16dimension*.ti,ab,kw.
33	16-d.ti,ab,kw.
34	16d.ti,ab,kw.
35	31 or 32 or 33 or 34
36	("quality of life" or qol or hrqol or hr-qol or utility or utilities or preference*).ti,ab,kw.
37	35 and 36
38	17-dimension*.ti,ab,kw.
39	17dimension*.ti,ab,kw.
40	17-d.ti,ab,kw.
41	17d.ti,ab,kw.
42	38 or 39 or 40 or 41
43	("quality of life" or qol or hrqol or hr-qol or utility or utilities or preference*).ti,ab,kw.
44	42 and 43
45	ahum.ti,ab,kw.
46	a-hum.ti,ab,kw.
47	adolescent health utility measure*.ti,ab,kw.
48	adolescent hum.ti,ab,kw.
49	45 or 46 or 47 or 48
50	eq-5d-y*.ti,ab,kw.
51	eq-5dy*.ti,ab,kw.
52	eq-5d-5l-y.ti,ab,kw.
53	eq-5d5ly.ti,ab,kw.
54	european quality of life 5d youth.ti,ab,kw.
55	european quality of life 5-d youth.ti,ab,kw.
56	european quality of life 5 dimension* youth.ti,ab,kw.
57	euroqol 5dy.ti,ab,kw.
58	euroqol 5d-y.ti,ab,kw.
59	euroqol 5 dimension* youth*.ti,ab,kw.
60	euroqol 5d youth*.ti,ab,kw.
61	euroqol 5-d youth.ti,ab,kw.
62	50 or 51 or 54 or 55 or 56 or 57 or 58 or 59 or 60 or 61
63	iqi.ti,ab,kw.
64	infant health related quality of life instrument*.ti,ab,kw.
65	infant quality of life instrument.ti,ab,kw.
66	infant qol instrument.ti,ab,kw.
67	infant hrqol instrument*.ti,ab,kw.
68	infant hr-qol instrument.ti,ab,kw.
69	tandi.ti,ab,kw.
70	("toddler and infant" adj2 "health related quality of life").ti,ab,kw.
71	("toddler and infant" adj2 hrqol).ti,ab,kw.
72	("toddler and infant" adj2 hr-qol).ti,ab,kw.
73	("Toddler and Infant" adj3 "european quality of life").ti,ab,kw.
74	("Toddler and Infant" adj3 euroqol).ti,ab,kw.
75	(eq-tips or eqtips).ti,ab,kw.

76	63 or 64 or 65 or 66 or 67 or 68 or 69 or 70 or 71 or 72 or 73 or 74 or 75
77	chscs-ps.ti,ab,kw.
78	comprehensive health status classification system for preschool children.ti,ab,kw.
79	comprehensive health status classification system for pre-school children.ti,ab,kw.
80	77 or 78 or 79
81	ch-6d.ti,ab,kw.
82	ch6d.ti,ab,kw.
83	ch 6dimension*.ti,ab,kw.
84	ch 6 dimension*.ti,ab,kw.
85	child health 6d*.ti,ab,kw.
86	child health 6-d*.ti,ab,kw.
87	81 or 82 or 83 or 84 or 85 or 86
88	quality of wellbeing.ti,ab,kw.
89	quality of well-being.ti,ab,kw.
90	qwb.ti,ab,kw.
91	88 or 89 or 90
92	12 or 19 or 30 or 37 or 44 or 49 or 62 or 76 or 80 or 87 or 91
93	premature birth/ or birth weight/
94	(preterm or pre-term or prematur*).ti,ab,kf.
95	("small for gestational age" or sga or (birthweight or birth weight or lbw* or vlbw* or elbw*)).ti,ab,kf.
96	93 or 94 or 95
97	92 and 96

Table S3 PsycINFO (OvidSP) 1806 to 26th April 2023

1	health utilities index.mp.
2	hui mark 2.mp.
3	hui2.mp.
4	hui-2.mp.
5	huiii.mp.
6	hui-ii.mp.
7	hui mark 3.mp.
8	hui3.mp.
9	hui-3.mp.
10	huiiii.mp.
11	hui-iii.mp.
12	1 or 2 or 3 or 4 or 5 or 6 or 7 or 8 or 9
13	child health utility 9d.mp.
14	child health utility 9-d.mp.
15	chu9d.mp.
16	chu-9d.mp.
17	child health utility 9-dimension*.mp.
18	child health utility 9dimension*.mp.
19	13 or 14 or 15 or 16 or 17 or 18
20	assessment of quality of life 6-dimension*.mp.
21	assessment of quality of life 6dimension*.mp.
22	assessment of quality of life 6-d.mp.
23	assessment of quality of life 6d.mp.
24	assessment of qol 6-dimension*.mp.
25	assessment of qol 6dimension*.mp.
26	assessment of qol 6-d.mp.
27	assessment of qol 6d.mp.
28	aqol-6d.mp.
29	aqol6d.mp.
30	20 or 21 or 22 or 23 or 24 or 25 or 26 or 27 or 28 or 29
31	16-dimension*.mp.
32	16dimension*.mp.
33	16-d.mp.
34	16d.mp.

35	31 or 32 or 33 or 34
36	("quality of life" or qol or hrqol or hr-qol or utility or utilities or preference*).mp.
37	35 and 36
38	17-dimension*.mp.
39	17dimension*.mp.
40	17-d.mp.
41	17d.mp.
42	38 or 39 or 40 or 41
43	("quality of life" or qol or hrqol or hr-qol or utility or utilities or preference*).mp.
44	42 and 43
45	ahum.mp.
46	a-hum.mp.
47	adolescent health utility measure*.mp.
48	adolescent hum.mp.
49	45 or 46 or 47 or 48
50	eq-5d-y*.mp.
51	eq-5dy*.mp.
52	eq-5d-5l-y.mp.
53	eq-5d5ly.mp.
54	european quality of life 5d youth.mp.
55	european quality of life 5-d youth.mp.
56	european quality of life 5 dimension* youth.mp.
57	euroqol 5dy.mp.
58	euroqol 5d-y.mp.
59	euroqol 5 dimension* youth*.mp.
60	euroqol 5d youth*.mp.
61	euroqol 5-d youth.mp.
62	50 or 51 or 54 or 55 or 56 or 57 or 58 or 59 or 60 or 61
63	iqi.mp.
64	infant health related quality of life instrument*.mp.
65	infant quality of life instrument.mp.
66	infant qol instrument.mp.
67	infant hrqol instrument*.mp.
68	infant hr-qol instrument.mp.
69	tandi.mp.
70	("toddler and infant" adj2 "health related quality of life").mp.
71	("toddler and infant" adj2 hrqol).mp.
72	("toddler and infant" adj2 hr-qol).mp.
73	("Toddler and Infant" adj3 "european quality of life").ti,ab,kw.
74	("Toddler and Infant" adj3 euroqol).ti,ab,kw.
75	(eq-tips or eqtips).ti,ab,kw.
76	63 or 64 or 65 or 66 or 67 or 68 or 69 or 70 or 71 or 72 or 73 or 74 or 75
77	chscs-ps.mp.
78	comprehensive health status classification system for preschool children.mp.
79	comprehensive health status classification system for pre-school children.mp.
80	77 or 78 or 79
81	ch-6d.mp.
82	ch6d.mp.
83	ch 6dimension*.mp.
84	ch 6 dimension*.mp.
85	child health 6d*.mp.
86	child health 6-d*.mp.
87	81 or 82 or 83 or 84 or 85 or 86
88	quality of wellbeing.mp.
89	quality of well-being.mp.
90	qwb.mp.
91	88 or 89 or 90
92	12 or 19 or 30 or 37 or 44 or 49 or 62 or 76 or 80 or 87 or 91
93	Premature Birth/ or Birth Weight/
94	(preterm or pre-term or prematur*).mp.

95	("small for gestational age" or sga or (birthweight or birth weight or lbw* or vlbw* or elbw*)).mp.
96	93 or 94 or 95
97	92 and 96
98	limit 97 to english language

Table S4 EconLit (Proquest) 1969 to 26th April 2023

**S12**	S10 AND S11
**S11**	noft(prematur* OR preterm OR pre-term OR "birth weight" OR birthweight OR lbw* OR vlbw* OR elbw* OR "small for gestational age" OR sga)
**S10**	S1 OR S2 OR S3 OR S4 OR S5 OR S5 OR S6 OR S7 OR S8 OR S9
**S9**	noft(chscs-p OR "comprehensive health status classification system for preschool children" OR "comprehensive health status classification system for pre-school children")
**S8**	noft(iqi OR "infant health related quality of life" OR "infant quality of life" OR "infant qol" OR "infant hrqol" OR "infant hr-qol" OR tandi) OR noft("toddler and infant" AND ("health related quality of life" OR hrqol OR hr-qol OR "european quality of life" OR euroqol)) OR noft(eq-tips OR eqtips)
**S7**	noft(("european quality of life" AND youth) OR (euroqol AND youth))
**S6**	noft(eq-5d-y* OR eq-5dy* OR eq-5d-yl-y OR eq-5dly)
**S5**	noft(ahum OR a-hum OR "adolescent health utility measure*" OR "adolescent hum")
**S4**	noft(16-dimension* OR 16dimension* OR 16d OR 16-d OR 17-dimension* OR 17dimension* OR 17d OR 17-d) AND noft("quality of life" or qol or hrqol or hr-qol or utility or utilities or preference*)
**S3**	noft("assessment of quality of life" OR "assessment of qol" OR aqol-6d OR aqol6d)
**S2**	noft("child health utility" OR chu-9d OR chu9d)
**S1**	noft("health utilities index" OR "hii mark 2" OR hui2 OR hui-2 OR huiii OR hui-ii OR "hui mark 3" OR hui3 OR hui-3 OR huiiii OR hui-iii)

Table S5 CINAHL (EBSCOHost) 1982 to 26th April 2023

S17	S12 AND S16 Limiters - English Language
S16	S13 OR S14 OR S15
S15	TI ((birthweight OR "birth weight" OR lbw* OR vlbw* OR elbw* OR "small for gestational age" OR sga) OR AB ((birthweight OR "birth weight" OR lbw* OR vlbw* OR elbw* OR "small for gestational age" OR sga)
S14	TI (preterm OR pre-term OR prematur*) OR AB (preterm OR pre-term OR prematur*)
S13	(MH "Infant, Low Birth Weight+") OR (MH "Infant, Premature") OR (MH "Outcomes of Prematurity")
S12	S1 OR S2 OR S3 OR S4 OR S5 OR S6 OR S7 OR S8 OR S9 OR S11
S11	TX "quality of wellbeing" OR "quality of well-being" OR qwb
S10	TX ch-6d OR ch6d OR "ch 6dimension*" OR "ch 6 dimension*" OR "child health 6d" OR "child health 6-d
S9	TX chscs-p OR "comprehensive health status classification system for preschool children" OR "comprehensive health status classification system for pre-school children"
S8	TX (("toddler and infant" N3 ("health related quality of life" OR hrqol OR hr-qol OR "european quality of life" OR euroqol))) OR TX (((iqi OR "infant health related quality of life" OR "infant quality of life" OR "infant qol" OR "infant hrqol" OR "infant hr-qol") N3 Instrument) OR tandi)) OR TX(eq-tips OR eqtips)
S7	TX (("european quality of life" N3 youth) OR (euroqol N3 youth))
S6	TX (eq-5d-y* OR eq-5dy* OR eq-5d-yl-y OR eq-5dly)
S5	TX (ahum OR "adolescent health utility measure*" OR "adolescent hum")
S4	TX (16-dimension* OR 16dimension* OR 16d OR 16-d OR 17-dimension* OR 17dimension* OR 17d OR 17-d) AND ("quality of life" or qol or hrqol or hr-qol or utility or utilities or preference*)
S3	TX((("assessment of quality of life" OR "assessment of qol") N2 (6dimension* OR "6 dimension*")) OR aqol-6d OR aqol6d)
S2	TX ("child health utility" OR chu-9d OR chu9d)
S1	TX ("health utilities index" OR "hii mark 2" OR hui2 OR hui-2 OR huiii OR hui-ii OR "hui mark 3" OR hui3 OR hui-3 OR huiiii OR hui-iii)

Table S6 Science Citation Index (Web of Science Core Collection) 1945 to 26th April 2023

15	#13 AND #12 Editions: WOS.SCI Limited to English
14	#13 AND #12 Editions: WOS.SCI
13	TS=(prematur* OR preterm OR pre-term) OR TS=("birth weight" OR birthweight OR lbw* OR vlbw* OR elbw* OR "small for gestational age" OR SGA) Editions: WOS.SCI
12	#11 OR #10 OR #9 OR #8 OR #7 OR #6 OR #5 OR #4 OR #3 OR #2 OR #1 Editions: WOS.SCI
11	TS=("quality of wellbeing" OR "quality of well-being" OR qwb) Editions: WOS.SCI
10	TS=(ch-6d OR ch6d OR "ch 6dimension*" OR "ch 6 dimension*" OR "child health 6d" OR "child health 6-d") Editions: WOS.SCI
9	TS=(chscs-p OR "comprehensive health status classification system for preschool children" OR "comprehensive health status classification system for pre-school children") Editions: WOS.SCI
8	TS=(iqi OR (("infant health related quality of life" OR "infant quality of life" OR "infant qol" OR "infant hrqol" OR "infant hr-qol") NEAR/3 Instrument) OR tandi) Editions: WOS.SCI
7	TS=("toddler and infant" NEAR/3 ("health related quality of life" OR hrqol OR hr-qol OR "european quality of life" OR euroqol)) OR TS=(eq-tips OR eqtips) Editions: WOS.SCI
6	TS=(eq-5d-y* OR eq-5dy* OR eq-5d-yl-y OR eq-5dly) OR TS=(("european quality of life" NEAR/3 youth) OR (euroqol NEAR/3 youth)) Editions: WOS.SCI
5	TS=(ahum OR "adolescent health utility measure*" OR "adolescent hum") Editions: WOS.SCI
4	TS=((16-dimension* OR 16dimension* OR 16d OR 16-d OR 17-dimension* OR 17dimension* OR 17d OR 17-d) AND ("quality of life" or qol or hrqol or hr-qol or utility or utilities or preference*)) Editions: WOS.SCI
3	TS=((("assessment of quality of life" OR "assessment of qol") NEAR/2 (6dimension* OR "6 dimension*")) OR aqol-6d OR aqol6d) Editions: WOS.SCI
2	TS=("child health utility" OR chu-9d OR chu9d) Editions: WOS.SCI
1	TS=("health utilities index" OR "hii mark 2" OR hui2 OR hui-2 OR huiii OR hui-ii OR "hui mark 3" OR hui3 OR hui-3 OR huiiii OR hui-iii) Editions: WOS.SCI

Table S7 Scopus (Elsevier) 1945 to 26th April 2023

1	(((TITLE-ABS-KEY ("health utilities index" OR "hui mark 2" OR hui2 OR hui-2 OR huiii OR hui-ii OR "hui mark 3" OR hui3 OR hui-3 OR huiiii OR hui-iii)) OR (TITLE-ABS-KEY ("child health utility" OR chu-9d OR chu9d)) OR (TITLE-ABS-KEY ((("assessment of quality of life" OR "assessment of qol") W/2 (6dimension* OR "6 dimension*")) OR aqol-6d OR aqol6)) OR (TITLE-ABS-KEY ((16-dimension* OR 16dimension* OR 16d OR 16-d OR 17-dimension* OR 17dimension* OR 17d OR 17-d) AND ("quality of life" OR qol OR hrqol OR hr-qol OR utility OR utilities OR preference*))) OR (TITLE-ABS-KEY (ahum OR "adolescent health utility measure*" OR "adolescent hum")) OR (TITLE-ABS-KEY (eq-5d-y* OR eq-5dy* OR eq-5d-yl-y OR eq-5dly)) OR (TITLE-ABS-KEY (("european quality of life" W/3 youth) OR (euroqol W/3 youth))) OR (TITLE-ABS-KEY (iqi OR (("infant health related quality of life" OR "infant quality of life" OR "infant qol" OR "infant hrqol" OR "infant hr-qol") W/3 instrument) OR tandi)) OR (TITLE-ABS-KEY (chscs-p OR "comprehensive health status classification system for preschool children" OR "comprehensive health status classification system for pre-school children")))) OR (TITLE-ABS-KEY ("toddler and infant" W/3 ("health related quality of life" OR hrqol OR hr-qol OR "european quality of life" OR euroqol)) OR TITLE-ABS-KEY (eq-tips OR eqtips))
2	(TITLE-ABS-KEY (prematur* OR preterm OR pre-term OR "birth weight" OR birthweight OR lbw* OR vlbw* OR elbw* OR "small for gestational age" OR sga))
3	1 AND 2

Evaluated psychometric properties

Table S8 Definitions of psychometric properties assessed by the systematic review and their performance criteria rating.

**Psychometric property (label)**[a]	**Definition**	**Criteria rating output**	**Criteria**[a,b]
**1. Reliability**			
1.1 Internal consistency (IC) [1-7]	The degree of the interrelatedness among items from the same scale.	+	Cronbach alpha for summary scores ≥0.7 AND item-total correlation ≥0.2 if both reported [7].
		±	Mixed assessment results: e.g., Cronbach alpha for summary scores ≥0.7 but item-total correlation <0.2 if both reported.
		-	Cronbach alpha for summary scores <0.7 AND item-total correlation <0.2 if both reported.
		?	Inconclusive results due to assessment design and method issues: e.g., small sample size [3].
1.2 Test-retest reliability (TR) [1-8]	The degree to which the instrument scores for patients are the same for repeated measurements over time, assuming no intervention or clinical change.	+	High agreement: e.g., intraclass correlation coefficient (ICC) ≥0.7 [5, 7].
		±	Mixed agreement results where multiple assessments conducted.
		-	Low agreement: e.g., ICC <0.7.
		?	Inconclusive results due to issues in assessment design and methods: e.g., inappropriate time interval, unclear whether health construct of interest remained stable over time interval [3].
1.3 Inter-rater reliability (IR) [1-4, 6-8]	The degree to which the instrument scores for patients are the same for ratings made by different (proxy) raters on the same occasion.	+	High agreement: e.g., ICC ≥0.7 [7].
		±	Mixed agreement results where multiple assessments conducted.
		-	Low agreement: e.g., ICC <0.7.
		?	Inconclusive results due to issues in assessment design and methods: e.g., unclear whether instrument applied to rater groups at similar time.
1.4 Inter-modal reliability (IM) [6, 8]	The degree to which the instrument scores for patients who have not changed are the same for measurements by different instrument administration modes (e.g., online, postal).	+	High agreement: e.g., ICC ≥0.7.
		±	Mixed agreement results where multiple assessments conducted.
		-	Low agreement: e.g., ICC <0.7.
		?	Inconclusive results due to issues in assessment design and methods: e.g., unclear whether instrument applied to modal groups at similar time.
**2. Proxy-child agreement (PC)** [9]	The extent of agreement in instrument scores between proxy respondent (e.g., parent) and child, where some discrepancies in measurement are expected due to different perspectives on childhood health.	+	High agreement: e.g., ICC ≥0.7 [7].
		±	Mixed agreement results where multiple assessments conducted.
		-	Low agreement: e.g., ICC <0.7.
		?	Inconclusive results due to issues in assessment design and methods: e.g., unclear whether the proxy is sufficiently aware of child's health status, excessively different administration mode between child and proxy
**3. Content validity (CV)** [1-9]	The degree to which the content of the instrument is an adequate reflection of the construct to be measured.	+	Conducted the following steps in original instrument development: (1) Stated the conceptual framework for the purpose of measurement. (2) Qualitative research with children or appropriate proxies. (3) Cognitive interviews and pilot tests with children or appropriate proxies [3, 5, 7, 9].[c]
		±	Conducted two of the three steps above.
		-	Conducted one or none of the three steps above; OR other evidence that the instrument content does not reflect the construct to be measured.
		?	Contained insufficient descriptions of the above assessments.

**4. Structural validity (SV)** [1-3, 7]	The degree to which the within-scale item relationships are an adequate reflection of the dimensionality of the construct to be measured by the scale.	+	Exploratory factor analysis: items with factor-loading coefficient ≥0.4 AND at least moderate correlations between scale scores (if both assessed) [7].
		±	Mixed assessment results from factor analysis.
		-	Exploratory factor analysis: items with factor-loading coefficient <0.4 AND low correlations between scale scores (if both assessed).
		?	Inconclusive results due to issues in assessment design and methods: e.g., unclear rotation method.
**5. Cross-cultural validity (CCV)** [1-3, 5, 6]	The degree to which the performance of the items on a translated or culturally adapted instrument are an adequate reflection of the performance of the items of the original version of the instrument.	+	Rigorous translation process: expert involvement; independent forward and backward translations; committee review; comparison to original (conceptual and linguistic equivalence); pre-testing (e.g., cognitive interviews). Should be followed by psychometric assessment of the cross-cultural version and comparable result to the original version [3, 6].
		±	Mixed translation process: e.g., expert involvement but no pre-testing.
		-	Problematic translation process and/or poor psychometric performance compared to the original version
		?	Provided insufficient detail on translation process to reach conclusion: e.g., insufficient description of the pre-test sample [3].
**6. Construct validity [6]**			
6.1 Known-group validity (KV) [1-5, 7]	The degree to which the instrument scores can differentiate groups with expected differences in the constructs measured by the instrument.	+	Description of subgroups delineated by clinical or sociodemographic variables and *a priori* hypotheses on instrument score differences. When score difference was tested between PLB group and normal term/BW controls without an *a priori* hypothesis, it was assumed that lower score is expected for PLB group. Statistically and clinically significant results consistent with hypotheses [7].[d,e,f]
		±	*A priori* hypotheses and mixed results where multiple between-group comparisons conducted.
		-	*A priori* hypotheses and results contrary to hypotheses.
		?	No *a priori* hypothesis that can interpret the significant/non-significant results or inconclusive due to assessment design and method issues.[g]
6.2 Hypothesis testing (HT) [1-3, 5]	The degree to which the instrument scores are associated with sociodemographic, clinical, and other variables according to hypotheses.	+	Description of variables potentially associated with instrument score and *a priori* hypotheses on strength and direction of association. Statistically and clinically significant result consistent with hypotheses [3, 5].[f]
		±	*A priori* hypotheses and mixed results where multiple associations examined.
		-	*A priori* hypotheses and results contrary to hypotheses.
		?	No *a priori* hypothesis that can interpret the significant/non-significant results or inconclusive results due to assessment design and method issues.[g]
6.3 Convergent validity (CNV) [1-4, 7]	The degree to which the instrument scores are correlated with other measures of the same or similar constructs.	+	*A priori* hypotheses and results consistent with hypotheses in terms of correlation strength (e.g., Pearson correlation coefficient >0.4),[h] statistical significance, and direction [7].
		±	*A priori* hypotheses and mixed results where multiple correlations assessed.
		-	*A priori* hypotheses and results contrary to hypotheses.
		?	No *a priori* hypothesis that can interpret the correlation or inconclusive results due to assessment design and method issues: e.g., statistical significance of correlation not reported.
6.4 Discriminant validity (DV) [1-4, 7]	The degree to which the instrument scores are not correlated with measures of different constructs.	+	*A priori* hypotheses and consistent results in terms of correlation strength (e.g., Pearson correlation coefficient <0.4),[h] statistical significance, and direction [7].
		±	*A priori* hypotheses and mixed results where multiple correlations assessed.
		-	*A priori* hypotheses and results contrary to hypotheses.
		?	No *a priori* hypothesis that can interpret the significant/non-significant results or inconclusive results due to assessment design and method issues.
		+	*A priori* hypotheses and consistent results in terms of clinically and statistically significant associations.

6.5 Empirical validity (EV) [8]	The degree to which the utility values generated by the preference-based instruments reflect people's preferences over health (e.g., self-reported health status)	±	*A priori* hypotheses and mixed assessment results where multiple associations assessed.
		-	*A priori* hypotheses and results contrary to hypotheses.
		?	No *a priori* hypothesis that can interpret the significant/non-significant results or inconclusive results due to assessment design and method issues.[i]
**7. Criterion-related validity [5, 6]**			
7.1 Concurrent validity (CRV) [1-4, 7]	The degree to which the instrument scores adequately reflect a "gold standard" criterion measured at the same time, i.e., correlation of scores with those of a criterion measure.	+	Described the 'gold standard' criterion and results consistent with *a priori* hypotheses in terms of correlation strength and direction between instrument and criterion scores [3, 7].
		±	*A priori* hypotheses and mixed assessment results where multiple correlations assessed.
		-	*A priori* hypotheses and results contrary to hypotheses.
		?	No *a priori* hypothesis that can interpret the significant/non-significant results or inconclusive results due to assessment design and method issues.[j]
7.2 Predictive validity (PV) [1-4, 7]	The degree to which the instrument scores adequately reflect a "gold standard" criterion measured in the future.	+	Described the 'gold standard' criterion and results consistent with *a priori* hypotheses in terms of associations between predicted instrument and criterion scores [3, 7].
		±	*A priori* hypotheses and mixed assessment results where multiple predicted associations assessed.
		-	*A priori* hypotheses and results contrary to hypotheses.
		?	No *a priori* hypothesis that can interpret the significant/non-significant results or inconclusive results due to assessment design and method issues: e.g., flaws in statistical method of prediction
**8. Responsiveness (RE)** [1-7]	The extent to which the instrument can identify differences in scores over time in individuals or groups who have changed with respect to the measurement concept.	+	Change in the instrument score consistent with: (i) *a priori* hypotheses on direction and strength of score change in response to a change of interest (e.g., intervention); and (ii) direction and strength of change in reference (e.g., disease-specific) measure [3, 4, 6, 7]. If the minimal clinically important change (MIC) is specified and justified, and score change is greater than MIC, then consistency with (ii) is not required.
		±	Mixed assessment results: e.g., consistent with changes in one reference measure but not with another.
		-	Score changes that are contrary to *a priori* hypotheses and/or change in the reference measure(s).
		?	No *a priori* hypothesis or reference measure that can interpret the significant/non-significant results or inconclusive due to assessment design and method issues: e.g., randomisation bias, inadequate power, unclear time interval between measurements, unclear description of intervention and expected impact [3].
**9. Acceptability (AC)** [5-8]	The level of data quality, assessed by its completeness and score distributions.	+	Conducted at least two assessments and met two or more of the following criteria: (i) missing data for instrument scores <5%;[k] (ii) floor and ceiling effects <10%;[l] (iii) high score distribution (e.g., number of unique health states >0.4 per respondent); (iv) other criteria concerning the ease of use (e.g., response time of <15 minutes) [6, 7].
		±	Conducted at least two assessments and met one of the four criteria above.
		-	Conducted at least two assessments and met none of the four criteria above.
		?	Conducted less than two assessments or unclear how many of the criteria are met due to poor reporting.[k]
**10. Interpretability (ITR)** [1-3, 5, 6]	The degree to which one can assign qualitative meaning (i.e., clinical or commonly understood connotations) to the instrument scores and change in scores and/or quantitative descriptions of minimally important difference (MID).	+	Conducted one of the following: (i) established instrument score mean and standard deviation for the normative reference population; (ii) quantified MIC or MID [3, 5].
		±	Compared instrument scores to external reference population scores (not necessarily established population norms) and/or to MIC/MID derived from external studies rather than primary derivation.
		?	Inconclusive results due to poor reporting or comparison features.
**Abbreviation:** ICC: intraclass correlation coefficient; MIC: minimal clinically important change; MID: minimal clinically important difference.			

[a] For all psychometric properties, sample size, low missing data, and appropriate statistical techniques were considered in evaluating the quality of assessment design and methods [3]. References to guidelines are provided alongside each label in the first column; these guidelines discussed the given property in terms of its definition and its relevance to determining an instrument's scientific credibility.
[b] Studies frequently contained multiple sub-assessments within the property assessment (e.g., KV assessment using multiple subgroup delineators), each of which received a criteria rating output. For a mix of '+' and '±' outputs, the higher output '+' was presented as the summary and likewise '±' for a mix of '±' and '-'. A mix of '+' and '-' was presented as '±'. A mix of '+'/'±'/'-' and '?' was presented as '+'/'±'/'-': i.e., an assessment received '?' only if *all* sub-assessments had issues in design and methods. References to the guidelines that offer specific criteria for the evaluation of psychometric performance is also provided.
[c] The review also considered CV evidence from non-original development studies. If so, reasonable criteria formulated by the primary study authors were used for evaluation.
[d] Few studies pre-specified *which* dimensions were expected to differ across groups beyond the general hypothesis that some dimension-level differences were expected. In this case, if ≥75% of dimensions (e.g., four or five dimensions out of five in EQ-5D-Y) showed significant differences, then '+' was given; <75% and >25% '±'; and ≤25% '-'.
[e] Where the study used Bonferroni correction for multiple comparisons (e.g., comparisons for multiple dimensions), the adjusted statistical significance threshold was used for evaluation.
[f] *Adjusted* between-group comparisons through multivariate regression models were evaluated under HT; only unadjusted comparisons under KV.
[g] Unadjusted comparison between sociodemographic subgroups without *a priori* statement of expected difference was evaluated under HT and given '?' for the *ad hoc* hypothesis testing.
[h] Different threshold was used if specified as part of *a priori* hypothesis [5].
[i] Where it was uncertain whether the subgroup delineator reflected people's preference over health, this assessment was evaluated under KV or HT.
[j] If there was insufficient justification for a measure (other than the instrument) being a 'gold standard' criterion, the assessment was evaluated under CNV.
[k] Effort was made to distinguish between missing data due to poor survey design (i.e., low response rate) from that due to the instrument itself (i.e., missing response from survey participants). Where this distinction was not possible due to poor reporting, the assessment was given '?'.
[l] Ceiling and floor effects at dimension level were not evaluated. Hence, ceiling/floor effect meant top/bottom level on all dimensions.

Excluded studies

Table S9 Studies excluded at full-text screening stage.

First author, Publication year	Title	Primary reason for exclusion
Martini, 2019	Quality of life of prematurely born schoolchildren; the caregiver's report and the child's self-report.	Not English full text
McNamara, 2012	The STOPPIT baby follow-up study: The effects of prophylactic in utero progesterone, given in twin pregnancy for the prevention of preterm birth, on child health and development at three to six years of age	No assessment of psychometric properties
McNamara, 2015	STOPPIT Baby Follow-up Study: the effect of prophylactic progesterone in twin pregnancy on childhood outcome	Not preterm population
Nomura, 2007	Conjoined effects of low birth weight and childhood abuse on adaptation and well-being in adolescence and adulthood	Did not use childhood instrument
Peart, 2020	Quality of life in children born extremely preterm/extremely low birth weight: 1991 to 2005	Full text reference already included
Rae, 2017	Creating a preference-based scoring system for the health status classification system-preschool (HSCS-PS)	Not preterm population
Roberts, 2011	Parent-reported health and wellbeing in extremely preterm and extremely low birth weight children at age 8 compared with term controls	Full text reference already included
van der Pal, 2012	Changes in quality of life into adulthood after very preterm birth and/or very low birth weight in the Netherlands	Full text reference already included
van der Pal-de Bruin, 2015	Profiling the preterm or VLBW born adolescent: Implications of the Dutch POPS cohort follow-up studies	No assessment of psychometric properties

Cohorts with multiple included primary studies

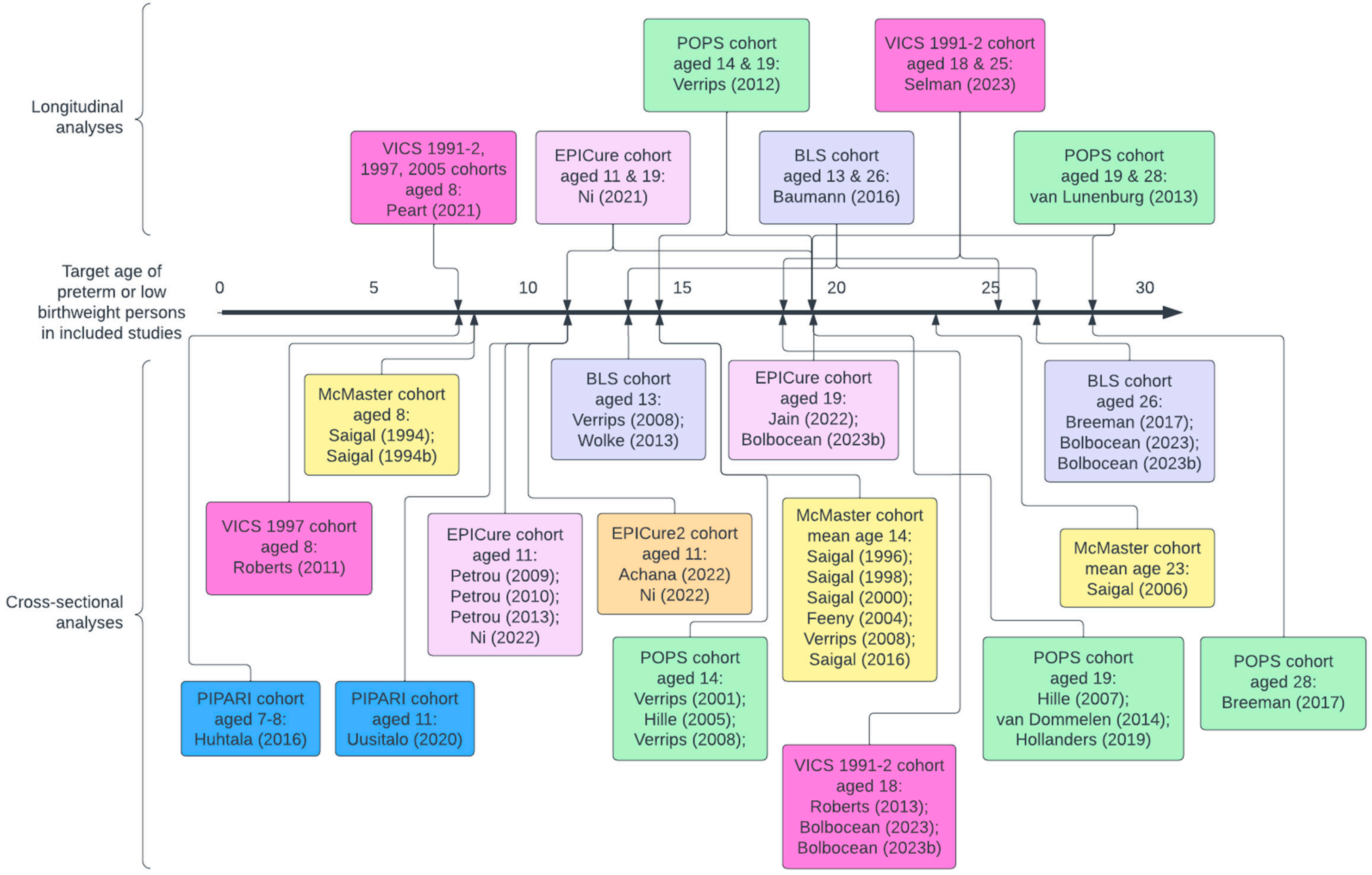

Figure S2 Cohorts of preterm or low birthweight persons which were analysed by multiple included studies and target age of studies.

Psychometric evaluation results

Table S10 Evaluation results for psychometric evidence from included studies by instrument.

Psychometric property abbreviations: IC: internal consistency; TR: test-retest reliability; IR: inter-rater reliability; IM: inter-modal reliability; PC: proxy-child agreement; CV: content validity; SV: structural validity; CCV: cross-cultural validity; KV: known-group validity; HT: hypothesis testing; CNV: convergent validity; DV: discriminant validity; EV: empirical validity; CRV: concurrent validity; PV: predictive validity; RE: responsiveness; AC: acceptability; ITR: interpretability.

#	Reference	Component (value set) [Language]	Resp. type	IC	TR	IR	IM	PC	CV	SV	CCV	KV	HT	CNV	DV	EV	CRV	PV	RE	AC	ITR
**17D**																					
1	Huhtala 2016 [10]	Index (Fin parent) [Fin/Swe]	PLB									+									
		Dimension [Fin/Swe]										+									
2	Rautava 2009 [11]	Index (Fin parent) [En]	Proxy										±								±
		Dimension [En]											±								
3	Uusitalo 2020 [12]	Index (Fin parent) [Fin/Swe]	PLB									±	-								±
		Dimension [Fin/Swe]										-								?	±
**CHSCS-PS**																					
1	Saigal 2005 [13]	Dimension [NS]	Proxy		±	±			+			+	+				+			+	
**HUI2**																					
1	Achana 2022 [14]	Index (UK adult) [En]	Proxy									+	+								
		Index (UK statistical inference) [En]										+	+								
		Index (Can parent) [En]										+	+								
		Dimension [En]										?								?	
2	Feeny 2004 [15]	Index (Can parent) [En]	PLB									+		±						?	±
3	Greenough 2004 [16]	Index (Can parent) [En]	Proxy									+									
4	James 2003 [17]	Index (NS) [En]	PLB									-									
		Dimension [En]										±								?	
5	Liu 2021 [18]	Index (NS) [En]	Proxy									±	±								
		Dimension [En]										±									
6	Peart 2021 [19]	Index (NS) [En]	Proxy									+								?	±
7	Petrou 2010 [20]	Index (UK adult) [En]	Proxy									+	±							?	
8	Petrou 2013 [21]	Index (UK adult) [En]	Proxy									+	+								
9		Index (NS) [En]	Proxy									+	±								

	Roberts 2011 [22]	Dimension [En]										+	±								
10	Saigal 1994 [23]	Dimension [NS]	Proxy						-			+								±	
11	Saigal 1994b [24]	Index (Can parent) [NS]	Proxy									+	?								
12	Saigal 1996 [25]	Index (Study children) [NS]	PLB									±									
		Dimension [NS]										±								-	
13	Saigal 1998 [26]	Dimension [NS]	PLB					+												±	
14	Saigal 2000 [27]	Index (Study parents) [NS]	Proxy									+									
		Dimension [NS]										±								-	
15	Saigal 2006 [28]	Dimension [NS]	PLB									+								±	
**HUI3**																					
1	Achana 2022 [14]	Index (Can adult) [En]	Proxy									+	+								
		Dimension [En]										?								?	
2	Baumann 2016 [29]	Index (NS) [NS]	PLB									-									
			Proxy									+									
		Dimension [NS]	PLB									±								-	
			Proxy									±								-	
3	Bolbocean 2023 [30]	Index (Can adult) [En]	PLB									+	+	-						?	±
4	Bolbocean 2023b [31]	Index (Can adult) [En]	PLB									+	+								±
		Dimension SAU (Can adult) [NS]											±								
		Dimension [NS]											+								
5	Breeman 2017 [32]	Index (Can adult) [NS]	PLB									+	-								±
		Dimension [NS]										±								±	
6	Feeny 2004 [15]	Index (Can adult) [En]	PLB									+		-						?	±
7	Gray 2007 [33]	Index (Can adult) [En]	PLB									-									
		Dimension [En]										-								?	
8	Greenough 2004 [16]	Index (NS) [En]	Proxy									-									
9	Greenough 2014 [34]	Index (NS) [En]	PLB									-									
			Proxy									-									
10	Hille 2005 [35]	Index (NS) [NS]	PLB									+								?	

11	Hille 2007 [36]	Dimension [NS]	PLB									+								?	
12	Hollanders 2019 [37]	Dimension [NS]	PLB									+	+							?	
13	Jain 2022 [38]	Vision dimension [EN]	PLB									-									
14	Ni 2021 [39]	Index (Can adult) [En]	Proxy																?		±
		Dimension [En]																	?		
15	Ni 2022 [40]	Index (Can adult) [En]	Proxy									+	+								±
		Dimension [En]										+								-	
16	Peart 2021 [19]	Index (NS) [En]	Proxy									+								?	±
17	Petrou 2009 [41]	Index (Can adult) [En]	Proxy									+	+								±
		Dimension [En]										+									
18	Petrou 2010 [20]	Index (Can adult) [En]	Proxy									±	±								±
		Dimension [En]										±									
19	Petrou 2013 [21]	Index (Can adult) [En]	Proxy									+	+								
		Dimension [En]										+									
20	Quinn 2004 [42]	Index (Can adult) [En]	Proxy									+									
		Dimension [En]										?								+	
21	Roberts 2013 [43]	Index (NS) [En]	PLB									+	+								
		Dimension SAU (NS) [EN]										+								?	
22	Saigal 2016 [44]	Index (Can adult) [NS]	PLB									+									±
		Dimension SAU (Can adult) [NS]										±								?	±
23	Selman 2023 [45]	Index (Can adult) [En]	PLB										-								±
		Dimension [En]											-								
24	van Dommelen 2014 [46]	Index (Can adult) [NS]	PLB										+								
25	van Lunenburg 2013 [47]	Index (Can adult) [NS]	PLB																?		±
		Dimension SAU (Can adult) [NS]																	?		
26	Verrips 2001 [48]	Index (Can adult) [Du]	PLB				-	±													±
		Unweighted sum [Du]					-	±													
		Dimension [Du]					-	-													
27	Verrips 2008 [49]	Index (Can adult) [NS]	PLB									+	+								±
		Dimension SAU (Can adult) [NS]										±	-							±	
28		Index (Can adult) [NS]	PLB										±						?		±

	Verrips 2012 [50]	Dimension SAU (Can adult) [NS]		?															?	-	
29	Wolke 2013 [51]	Index (Can adult) [Ger]	PLB									+	±								±
			Proxy					-				+	±								±
		Dimension [Ger]	PLB									±									
			Proxy					±				+									

Abbreviation: Du: Dutch; En: English; EP/ELBW: extremely preterm and/or extremely low birthweight; Ger: German; Fin: Finnish; NS: not stated; PLB: preterm or low birthweight; P/LBW: preterm and/or low birthweight; Resp.: respondent; SAU: single-attribute utility; Swe: Swedish; VP/VLBW: very preterm and/or very low birthweight

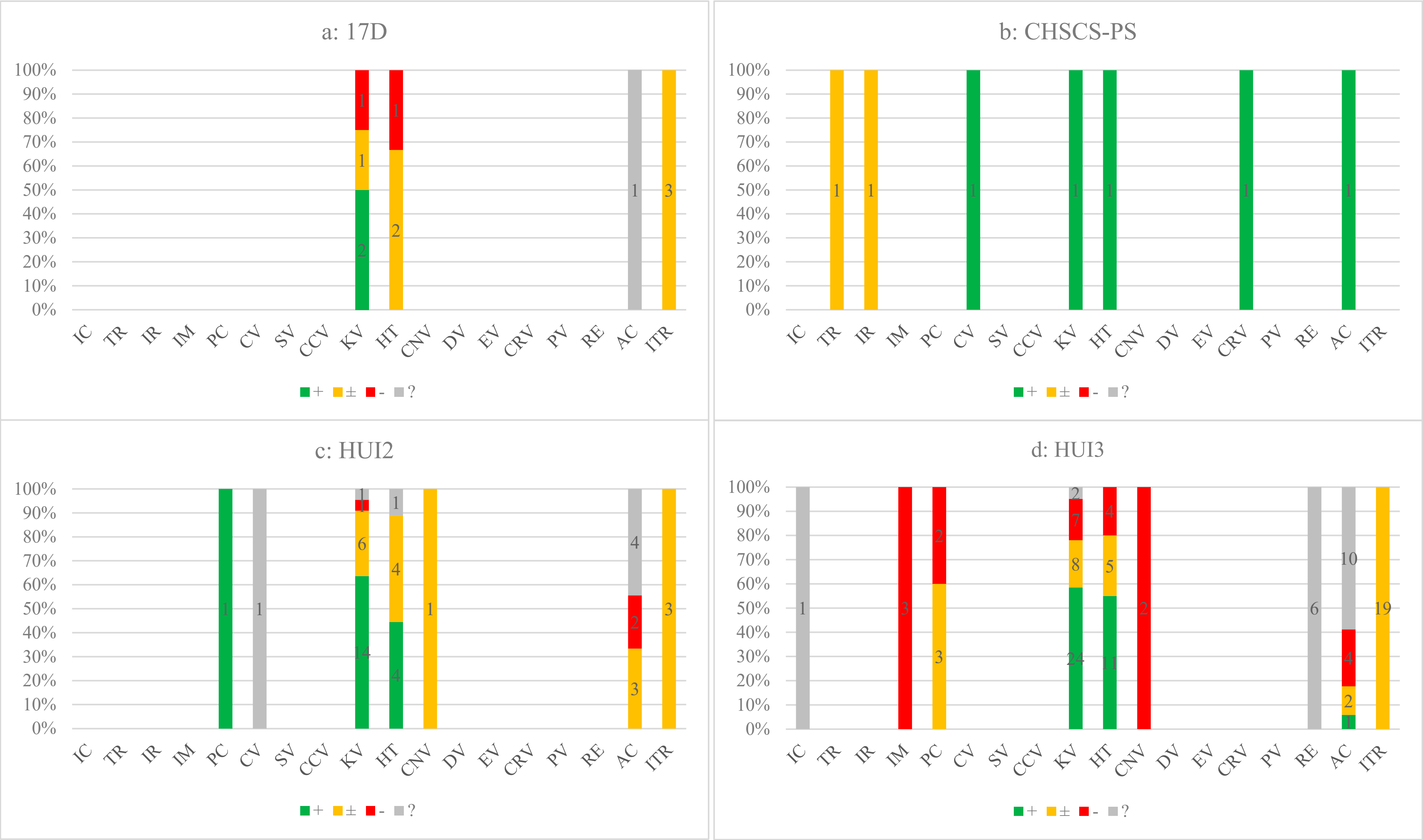

Figure S3 Criteria rating outputs by instrument and psychometric property. **Note:** absolute numbers of criteria rating outputs are displayed within each bar.

References

1. Mokkink LB, Terwee CB, Knol DL, Stratford PW, Alonso J, Patrick DL, et al. The COSMIN checklist for evaluating the methodological quality of studies on measurement properties: a clarification of its content. BMC medical research methodology. 2010;10(1):1-8.
2. Mokkink LB, Terwee CB, Patrick DL, Alonso J, Stratford PW, Knol DL, et al. The COSMIN study reached international consensus on taxonomy, terminology, and definitions of measurement properties for health-related patient-reported outcomes. Journal of clinical epidemiology. 2010;63(7):737-45.
3. Mokkink LB, Terwee CB, Patrick DL, Alonso J, Stratford PW, Knol DL, et al. The COSMIN checklist for assessing the methodological quality of studies on measurement properties of health status measurement instruments: an international Delphi study. Quality of life research. 2010;19(4):539-49.
4. Food and Drug Administration. Patient reported outcome measures: use in medical product development to support labelling claims. Washington DC. 2009.
5. Reeve BB, Wyrwich KW, Wu AW, Velikova G, Terwee CB, Snyder CF, et al. ISOQOL recommends minimum standards for patient-reported outcome measures used in patient-centered outcomes and comparative effectiveness research. Quality of Life Research. 2013;22(8):1889-905.
6. Lohr KN. Assessing health status and quality-of-life instruments: attributes and review criteria. Quality of life research. 2002;11(3):193-205.
7. Smith S, Lamping D, Banerjee S, Harwood R, Foley B, Smith P, et al. Measurement of health-related quality of life for people with dementia: development of a new instrument (DEMQOL) and an evaluation of current methodology. Health Technology Assessment (Winchester, England). 2005;9(10):1-iv.
8. Brazier J, Deverill M. A checklist for judging preference-based measures of health related quality of life: learning from psychometrics. Health economics. 1999;8(1):41-51.
9. Matza LS, Patrick DL, Riley AW, Alexander JJ, Rajmil L, Pleil AM, et al. Pediatric patient-reported outcome instruments for research to support medical product labeling: report of the ISPOR PRO good research practices for the assessment of children and adolescents task force. Value in Health. 2013;16(4):461-79.
10. Huhtala M, Korja R, Rautava L, Lehtonen L, Haataja L, Lapinleimu H, et al. Health-related quality of life in very low birth weight children at nearly eight years of age. Acta Paediatrica. 2016;105(1):53-9.
11. Rautava L, Häkkinen U, Korvenranta E, Andersson S, Gissler M, Hallman M, et al. Health-related quality of life in 5-year-old very low birth weight infants. The Journal of pediatrics. 2009;155(3):338-43. e3.
12. Uusitalo K, Haataja L, Nyman A, Ripatti L, Huhtala M, Rautava P, et al. Preterm children's developmental coordination disorder, cognition and quality of life: a prospective cohort study. BMJ paediatrics open. 2020;4(1).
13. Saigal S, Rosenbaum P, Stoskopf B, Hoult L, Furlong W, Feeny D, et al. Development, reliability and validity of a new measure of overall health for pre-school children. Quality of Life Research. 2005;14(1):243-52.
14. Achana F, Johnson S, Ni Y, Marlow N, Wolke D, Khan K, et al. Economic costs and health utility values associated with extremely preterm birth: Evidence from the EPICure2 cohort study. Paediatric and Perinatal Epidemiology. 2022;36(5):696-705.
15. Feeny D, Furlong W, Saigal S, Sun J. Comparing directly measured standard gamble scores to HUI2 and HUI3 utility scores: group- and individual-level comparisons. Soc Sci Med. 2004;58(4):799-809. Epub 2003/12/16. PubMed PMID: 14672594.
16. Greenough A, Alexander J, Burgess S, Bytham J, Chetcuti P, Hagan J, et al. Health care utilisation of prematurely born, preschool children related to hospitalisation for RSV infection. Archives of Disease in Childhood. 2004;89(7):673-8.
17. James JD. Health status and psychological adjustment in low birth weight and normal birth weight Jamaican preadolescents: Northwestern University; 2003.
18. Liu GX, Harding JE, Team PS. Caregiver-reported health-related quality of life of New Zealand children born very and extremely preterm. Plos one. 2021;16(6):e0253026.

19. Peart S, Cheong JLY, Roberts G, Davis N, Anderson PJ, Doyle LW. Changes over time in quality of life of school-aged children born extremely preterm: 1991–2005. Archives of Disease in Childhood-Fetal and Neonatal Edition. 2021;106(4):425-9.
20. Petrou S, Johnson S, Wolke D, Hollis C, Kochhar P, Marlow N. Economic costs and preference-based health-related quality of life outcomes associated with childhood psychiatric disorders. The British Journal of Psychiatry. 2010;197(5):395-404.
21. Petrou S, Johnson S, Wolke D, Marlow N. The association between neurodevelopmental disability and economic outcomes during mid-childhood. Child: care, health and development. 2013;39(3):345-57.
22. Roberts G, Anderson PJ, Cheong J, Doyle LW, Group VICS. Parent-reported health in extremely preterm and extremely low-birthweight children at age 8 years compared with comparison children born at term. Developmental Medicine & Child Neurology. 2011;53(10):927-32.
23. Saigal S, Rosenbaum P, Stoskopf B, Hoult L, Furlong W, Feeny D, et al. Comprehensive assessment of the health status of extremely low birth weight children at eight years of age: comparison with a reference group. The Journal of pediatrics. 1994;125(3):411-7.
24. Saigal S, Feeny D, Furlong W, Rosenbaum P, Burrows E, Torrance G. Comparison of the health-related quality of life of extremely low birth weight children and a reference group of children at age eight years. The Journal of pediatrics. 1994;125(3):418-25.
25. Saigal S, Feeny D, Rosenbaum P, Furlong W, Burrows E, Stoskopf B. Self-perceived health status and health-related quality of life of extremely low-birth-weight infants at adolescence. Jama. 1996;276(6):453-9.
26. Saigal S, Rosenbaum P, Hoult L, Furlong W, Feeny D, Burrows E, et al. Conceptual and methodological issues in assessing health-related quality of life in children and adolescents: illustration from studies of extremely low birthweight survivors. Measuring Health-Related Quality of Life in Children and Adolescents: Implications for Research and Practice Mahwah, NJ: Lawrence Erlbaum Associates. 1998:151-69.
27. Saigal S, Rosenbaum PL, Feeny D, Burrows E, Furlong W, Stoskopf BL, et al. Parental perspectives of the health status and health-related quality of life of teen-aged children who were extremely low birth weight and term controls. Pediatrics. 2000;105(3):569-74.
28. Saigal S, Stoskopf B, Pinelli J, Streiner D, Hoult L, Paneth N, et al. Self-perceived health-related quality of life of former extremely low birth weight infants at young adulthood. Pediatrics. 2006;118(3):1140-8.
29. Baumann N, Bartmann P, Wolke D. Health-related quality of life into adulthood after very preterm birth. Pediatrics. 2016;137(4).
30. Bolbocean C, Anderson PJ, Bartmann P, Cheong JL, Doyle LW, Wolke D, et al. Comparative evaluation of the health utilities index mark 3 and the short form 6D: evidence from an individual participant data meta-analysis of very preterm and very low birthweight adults. Quality of Life Research. 2023:1-14.
31. Bolbocean C, van der Pal S, van Buuren S, Anderson PJ, Bartmann P, Baumann N, et al. Health-related quality-of-life outcomes of very preterm or very low birth weight adults: Evidence from an individual participant data meta-analysis. PharmacoEconomics. 2023;41(1):93-105.
32. Breeman LD, van der Pal S, Verrips GH, Baumann N, Bartmann P, Wolke D. Neonatal treatment philosophy in Dutch and German NICUs: health-related quality of life in adulthood of VP/VLBW infants. Quality of Life Research. 2017;26:935-43.
33. Gray R, Petrou S, Hockley C, Gardner F. Self-reported health status and health-related quality of life of teenagers who were born before 29 weeks' gestational age. Pediatrics. 2007;120(1):e86-93. Epub 2007/07/04. doi: 10.1542/peds.2006-2034. PubMed PMID: 17606553.
34. Greenough A, Peacock J, Zivanovic S, Alcazar-Paris M, Lo J, Marlow N, et al. United Kingdom Oscillation Study: long-term outcomes of a randomised trial of two modes of neonatal ventilation. Health Technol Assess. 2014;18(41):v-xx, 1-95. Epub 2014/06/28. doi: 10.3310/hta18410. PubMed PMID: 24972254.
35. Hille E, Den Ouden A, Stuifbergen M, Verrips G, Vogels A, Brand R, et al. Is attrition bias a problem in neonatal follow-up? Early human development. 2005;81(11):901-8.
36. Hille EeT, Weisglas-Kuperus N, Van Goudoever J, Jacobusse GW, Ens-Dokkum MH, de Groot L, et al. Functional outcomes and participation in young adulthood for very preterm and very low birth

weight infants: the Dutch Project on Preterm and Small for Gestational Age Infants at 19 years of age. Pediatrics. 2007;120(3):e587-e95.
37. Hollanders JJ, Schaëfer N, van der Pal SM, Oosterlaan J, Rotteveel J, Finken MJ, et al. Long-term neurodevelopmental and functional outcomes of infants born very preterm and/or with a very low birth weight. Neonatology. 2019;115(4):310-9.
38. Jain S, Sim PY, Beckmann J, Ni Y, Uddin N, Unwin B, et al. Functional ophthalmic factors associated with extreme prematurity in young adults. JAMA Network Open. 2022;5(1):e2145702-e.
39. Ni Y, O'Reilly H, Johnson S, Marlow N, Wolke D. Health-related quality of life from adolescence to adulthood following extremely preterm birth. The Journal of Pediatrics. 2021;237:227-36. e5.
40. Ni Y, Johnson S, Marlow N, Wolke D. Reduced health-related quality of life in children born extremely preterm in 2006 compared with 1995: the EPICure Studies. Archives of Disease in Childhood-Fetal and Neonatal Edition. 2022;107(4):408-13.
41. Petrou S, Abangma G, Johnson S, Wolke D, Marlow N. Costs and health utilities associated with extremely preterm birth: evidence from the EPICure study. Value in Health. 2009;12(8):1124-34.
42. Quinn GE, Dobson V, Saigal S, Phelps DL, Hardy RJ, Tung B, et al. Health-related quality of life at age 10 years in very low-birth-weight children with and without threshold retinopathy of prematurity. Arch Ophthalmol. 2004;122(11):1659-66.
43. Roberts G, Burnett AC, Lee KJ, Cheong J, Wood SJ, Anderson PJ, et al. Quality of life at age 18 years after extremely preterm birth in the post-surfactant era. The Journal of pediatrics. 2013;163(4):1008-13. e1.
44. Saigal S, Ferro MA, Van Lieshout RJ, Schmidt LA, Morrison KM, Boyle MH. Health-related quality of life trajectories of extremely low birth weight survivors into adulthood. The Journal of pediatrics. 2016;179:68-73. e1.
45. Selman C, Mainzer R, Lee K, Anderson P, Burnett A, Garland SM, et al. Health-related quality of life in adults born extremely preterm or with extremely low birth weight in the postsurfactant era: a longitudinal cohort study. Archives of Disease in Childhood-Fetal and Neonatal Edition. 2023.
46. Van Dommelen P, Van Der Pal SM, Bennebroek Gravenhorst J, Walther FJ, Wit JM, van der Pal de Bruin KM. The effect of early catch-up growth on health and well-being in young adults. Annals of Nutrition and Metabolism. 2014;65(2-3):220-6.
47. van Lunenburg A, van der Pal SM, van Dommelen P, van der Pal–de Bruin KM, Bennebroek Gravenhorst J, Verrips GH. Changes in quality of life into adulthood after very preterm birth and/or very low birth weight in the Netherlands. Health and Quality of Life Outcomes. 2013;11:1-8.
48. Verrips G, Stuifbergen M, Den Ouden A, Bonsel G, Gemke R, Paneth N, et al. Measuring health status using the Health Utilities Index: agreement between raters and between modalities of administration. Journal of Clinical Epidemiology. 2001;54(5):475-81.
49. Verrips E, Vogels T, Saigal S, Wolke D, Meyer R, Hoult L, et al. Health-related quality of life for extremely low birth weight adolescents in Canada, Germany, and the Netherlands. Pediatrics. 2008;122(3):556-61.
50. Verrips G, Brouwer L, Vogels T, Taal E, Drossaert C, Feeny D, et al. Long term follow-up of health-related quality of life in young adults born very preterm or with a very low birth weight. Health and Quality of Life Outcomes. 2012;10(1):1-8.
51. Wolke D, Chernova J, Eryigit-Madzwamuse S, Samara M, Zwierzynska K, Petrou S. Self and parent perspectives on health-related quality of life of adolescents born very preterm. The Journal of pediatrics. 2013;163(4):1020-6. e2.
